# Five new species of Entomobryinae (Collembola, Entomobryidae) from China

**DOI:** 10.3897/zookeys.1275.158254

**Published:** 2026-03-26

**Authors:** Xiao-Wei Qian, Yi-Tong Ma

**Affiliations:** 1 School of Life Sciences, Nantong University, Nantong, Jiangsu, 226000, China School of Life Sciences, Nantong University Nantong China

**Keywords:** Chaetotaxy, identification key, *
Lepidodens
*, *
Lepidosira
*, springtails, taxonomy, *
Willowsia
*

## Abstract

Five new scaled species, belonging to three genera of Entomobryinae, are described from Guangxi Zhuang Autonomous Region and Chongqing Municipality, China. *Lepidodens maculata***sp. nov**. is characterised by small blue spots on the body; *Lepidosira cheni***sp. nov**., *L. guilinensis***sp. nov**., and *L. montis***sp. nov**. by their colour pattern and dorsal chaetotaxy of the body; and *Willowsia zhangi***sp. nov**. by its colour pattern. A key to the scaled genera of the subfamily Entomobryinae and a key to the species of *Lepidodens* are provided.

## Introduction

With the recent development of molecular biology and the discovery of new morphological characters, a great change has taken place in the classification of the family Entomobryidae. The tribe Willowsiini was abandoned (Zhang and Deharveng 2015), and the family Entomobryidae was divided into six subfamilies (Entomobryinae, Lepidocyrtinae, Paronellidinae, Paronellinae, Salininae, and Seirinae) (Godeiro et al. 2023). Among these six subfamilies, the largest, Entomobryinae, contains 31 genera; 15 genera are without scales on the body and 16 have scales. Among the scaled genera, the shape and distribution of scales are important in the generic taxonomy, separated by the following key.

### Key to the scaled genera of the subfamily Entomobryinae

**Table:** 

1	Abd. VI with finger-like projection	*** Epimetrura ***
–	Abd. VI without finger-like projection	**2**
2	Scales present on dens	**3**
–	Scales absent on dens	**7**
3	Dental spines present	*** Acanthocyrtus ***
–	Dental spines absent	**4**
4	Basal ribs of scales longer than distal ones	*** Lepidodens ***
–	Basal ribs of scales almost as long as distal ones	**5**
5	Scales narrow and tip pointed	*** Lepidobrya ***
–	Scales not narrow and tip not pointed	**6**
6	Manubrium with distal thick blunt mac	*** Lepidocyrtoides ***
–	Manubrium without distal thick blunt mac	*** Lepidosira ***
7	Dental spines present	**8**
–	Dental spines absent	**9**
8	Prelabral bifurcate	*** Amazhomidia ***
–	Prelabral not bifurcate	*** Sinhomidia ***
9	Eyes absent	**10**
–	Eyes present	**12**
10	Mucro bidentate	*** Szeptyckiella ***
–	Mucro falcate	**11**
11	Body mac well developed	*** Hawinella ***
–	Body mac strongly reduced	*** Lepidosinella ***
12	Mucro falcate	**13**
–	Mucro bidentate	**14**
13	Mucronal basal spine absent	*** Desertia ***
–	Mucronal basal spine present	*** Drepanosira ***
14	Scales chaeta-like; scales of posterior row of tergites strongly elongate	*** Janetschekbrya ***
–	Scales not chaeta-like; scales of posterior row of tergites not strongly elongate	**15**
15	Scales narrow and with 2 uninterrupted lateral ribs, mac reduced	*** Americabrya ***
–	Scales different types and mac as usual	*** Willowsia ***

There are two main morphological characters in the taxonomy of the genus *Lepidodens* Zhang & Pan, 2016: (1) scales are heavily striate with basal ribs longer than distal ones and present on the manubrium and dentes besides the trunk; and (2) ms on Abd. I is anterior to m_3_mac. Currently, the genus contains five species, all of which have been reported from China.

*Lepidosira* was established by Schött in 1925 for the species *L. montana* Schött, 1925. The main characters of the genus include scales on the manubrium and dentes, a retractile terminal organ on the apical antenna, and a bidentate mucro with a basal spine (Schött 1925). To date, 57 species of the genus have been described worldwide (Bellinger et al. 2025), ~ 40 of which are from Oceania, and the other species are from Brazil (1), Costa Rica (1), India (2), Indonesia (3), Japan (1), Vietnam (2), Rwanda, and Burundi (1). There were no reports of the genus from China.

Although scales are present on the body in the genus *Willowsia* Shoebotham, 1917, they are absent on the dentes. The scales of the genus were divided into four types: spinulate type, short rib type, long basal rib type, and an uninterrupted type (Zhang et al. 2011). Forty-six species have been described worldwide, ranging from the Arctic region to tropical areas, and approximately half are from China (Bellinger et al. 2025).

Here we describe one new species of *Lepidodens*, three new species of *Lepidosira*, and one new species of *Willowsia* from China and provide a key to the species of the genus *Lepidodens*.

## Materials and methods

Specimens were collected in two reserves: the Huaping National Nature Reserve and the Yintiaoling National Nature Reserve. The Huaping National Nature Reserve is located at northeast of the Guangxi Zhuang Autonomous Region, which belongs to South China. It has a subtropical humid monsoon climate with annual average temperature of about 13°C and an annual average precipitation of 2,200 mm. The Yintiaoling National Nature Reserve is located at northeast of the Chongqing Municipality, which belongs to Southwestern China. It has a subtropical humid monsoon climate with annual average temperature of ~ 10°C and an annual average precipitation of 1,500 mm. Specimens were collected with an aspirator and stored in 99 % alcohol. They were mounted on glass slides in Marc André II solution and examined using a Leica DM2500 phase contrast microscope. Photographs were taken using a Leica DFC300 FX digital camera mounted on the microscope and enhanced with Photoshop CS2 (Adobe Inc.). SEM photographs were taken under a Zeiss GeminiSEM 300 after the specimens were coated with a Leica EM ACE600. Type specimens were deposited in the School of Life Sciences, Nantong University, Jiangsu, China.

The nomenclature of the dorsal macrochaetotaxy of the head and interocular chaetae follows Jordana and Baquero (2005) and Mari-Mutt (1986). Labial chaetae are designated following Gisin (1964). Labral and tergal chaetae of the body follow Szeptycki (1973, 1979).

### Abbreviations

**Ant**. Antennal segment(s);

**Th**. Thoracic segment(s);

**Abd**. Abdominal segment(s);

**mac** macrochaeta(e);

**mes** mesochaeta(e);

**ms** specialized microchaeta(e);

**sens** specialised ordinary chaeta(e);

**Gr**. Group.

## Results

### Class Collembola Lubbock, 1873

**Order Entomobryomorpha Börner, 1913**

**Family Entomobryidae Tömösvary, 1882**

#### Genus Lepidodens

Taxon classificationAnimaliaEntomobryomorphaEntomobryidae

Zhang & Pan, 2016

1939A376-46EA-5A4C-81BC-68B7BBC58766

##### Type species.

*Lepidodens nigrofasciatus* Zhang & Pan, 2016: 602.

#### Lepidodens maculata sp. nov.

Taxon classificationAnimaliaEntomobryomorphaEntomobryidae

FDF4E2EC-86F4-5905-94AE-97EB1982685F

https://zoobank.org/2C08DF22-ED89-466B-974A-72557C4DDB76

Figs 1, 2, 3, 4, 5, 6, 7, 8, 9, 10, 11, 12, 13, 14, 15, 16, 17, 18, 19, 20, 21, 22, 23, 24, 25, 26, 27, 28, 29, 30, 31, 32, 33, 34, Tables 1, 2

##### Type material.

***Holotype*** • ♀ on slide, China, Guangxi Autonomous Region, Guilin City, Longsheng Autonomous County, Huaping Natural Reserve, Tianping Mountain, 31-V-2023, 25°37'52"N, 109°54'47"E, 935.4 m asl, sample number 1281. ***Paratypes*** • ♀ on slide, same data as holotype; • 2 ♀♀ on slides, China, Chongqing Municipality, Wuxi County, Yintiaoling National Nature Reserve, Hongqi Protection Station, Hundred-step Stair, 21-VII-2024, 31°29'01"N, 109°49'21"E, 1359.9 m asl, sample number 1307; • 2 ♀♀ on slides, China, Chongqing Municipality, Wuxi County, Yintiaoling National Nature Reserve, Linzikou Protection Station, 24-VII-2024, 31°28'27"N, 109°52'40"E, 1232.5 m asl, sample number 1314. All collected by Y-T Ma.

##### Description.

***Size***: Body length up to 2.80 mm.

**Figures 1, 2.**
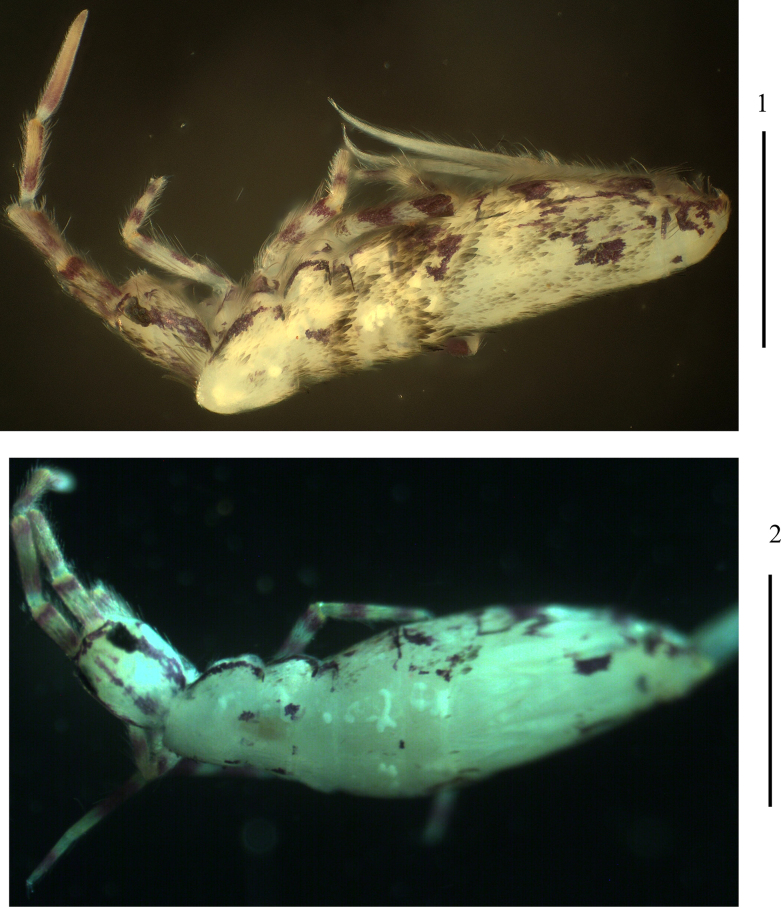
Habitus of *Lepidodens maculata* sp. nov. (**1**. Lateral view; **2**. Dorsal view). Scale bars: 1 mm.

***Colour pattern***: Ground colour pale yellow. Eye patches dark blue; basal and distal parts of each segment of Ant. I–III blue pigmented; dorsal head with a longitudinal blue stripe along midline and behind eyepatch, respectively; an irregular longitudinal blue stripe also presents on lateral sides from Th. II to Abd. III. Th. III, Abd. IV, and sometimes Th. II with a pair of small blue sublateral spots (Figs 1, 2).

***Scales***: Scales pointed with basal ribs longer than distal one (Figs 3, 4), present on terga (Fig. 5), Ant. I–II (Fig. 6), head (Fig. 7), legs (Fig. 8), ventral tube (Fig. 9), ventral side of manubrium and dentes (Figs 10, 11).

**Figures 3–11.**
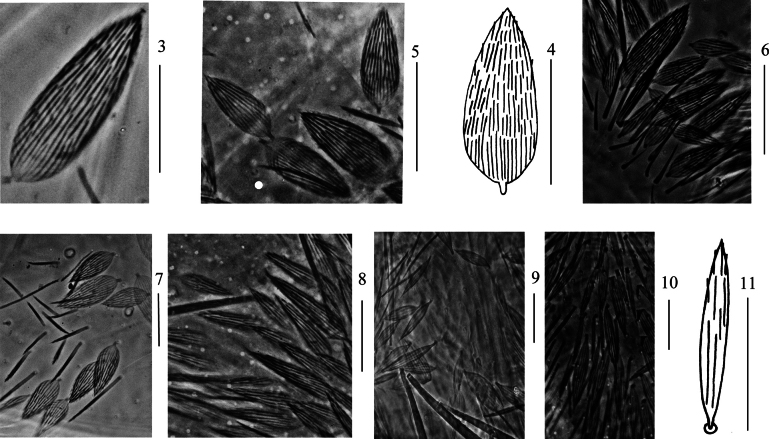
Scales of *Lepidodens maculata* sp. nov. **3**. Photomicrograph of scale (dorsal view); **4**. Scale (dorsal view); **5**. Photomicrograph of scales on terga (dorsal view); **6**. Photomicrograph of scales on Ant. I–II (dorsal view); **7**. Photomicrograph of scales on head (dorsal view); **8**. Photomicrograph of scales on leg; **9**. photomicrograph of scales on ventral tube (anterior view); **10**. photomicrograph of scales on ventral dens; **11**. scale on ventral dens. Scale bars: 20 μm.

**Figures 12–19.**
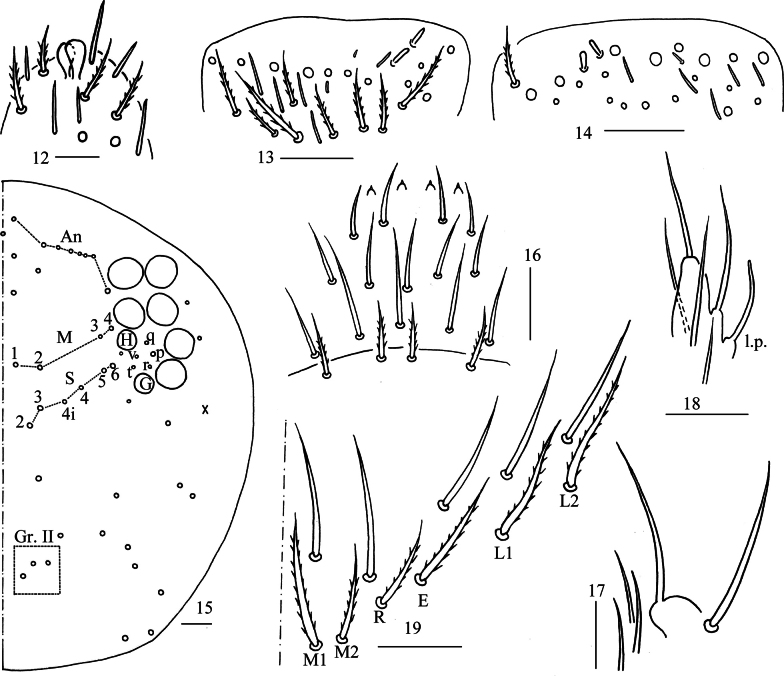
*Lepidodens maculata* sp. nov. **12**. apex of Ant. IV (dorsal view); **13**. distal Ant. III (ventral view); **14**. distal Ant. II (ventral view); **15**. dorsal head (right side); **16**. prelabrum and labrum (dorsal view); **17**. maxillary palp and outer lobe (right side); **18**. labial papilla E; **19**. labial and post-labial chaetotaxy (right side). Abbreviations: l. p. = lateral process, right side. Scale bars: 20 μm.

***Head***: Antenna not annulated and 0.48–0.65 times length of body. Ratio of Ant. I–IV as 1.00/1.20–1.78/1.10–1.57/1.76–2.73. Distal part of Ant. IV with many sensory chaetae and normal ciliate chaetae, apical bulb bilobed (Fig. 12). Sensory organ of Ant. III with two rod-like chaetae (Fig. 13). Ant. II with 2–3 rod-like sensilla apically (Fig. 14). Eyes 8+8, G and H smaller than others, interocular chaetae as p, q, v, r, t. Dorsal chaetotaxy of head with 7–8 (rarely 9) antennal (An), four median (M), six sutural (S) mac and three mac in Gr. II (Fig. 15). Prelabral and labral chaetae as 4/5, 5, 4, prelabral chaetae ciliate and other smooth, labral papillae conical (Fig. 16). Basal chaeta on maxillary outer lobe almost as thick as apical one; sublobal plate with three smooth chaetae-like processes (Fig. 17). Lateral process of labial papilla E differentiated, with tip reaching or slightly exceeding apex of papilla E (Fig. 18). Labial base with M_1_M_2_REL_1_L_2_, M_2_ sometimes absent, all ciliate (Fig. 19).

**Figures 20–25.**
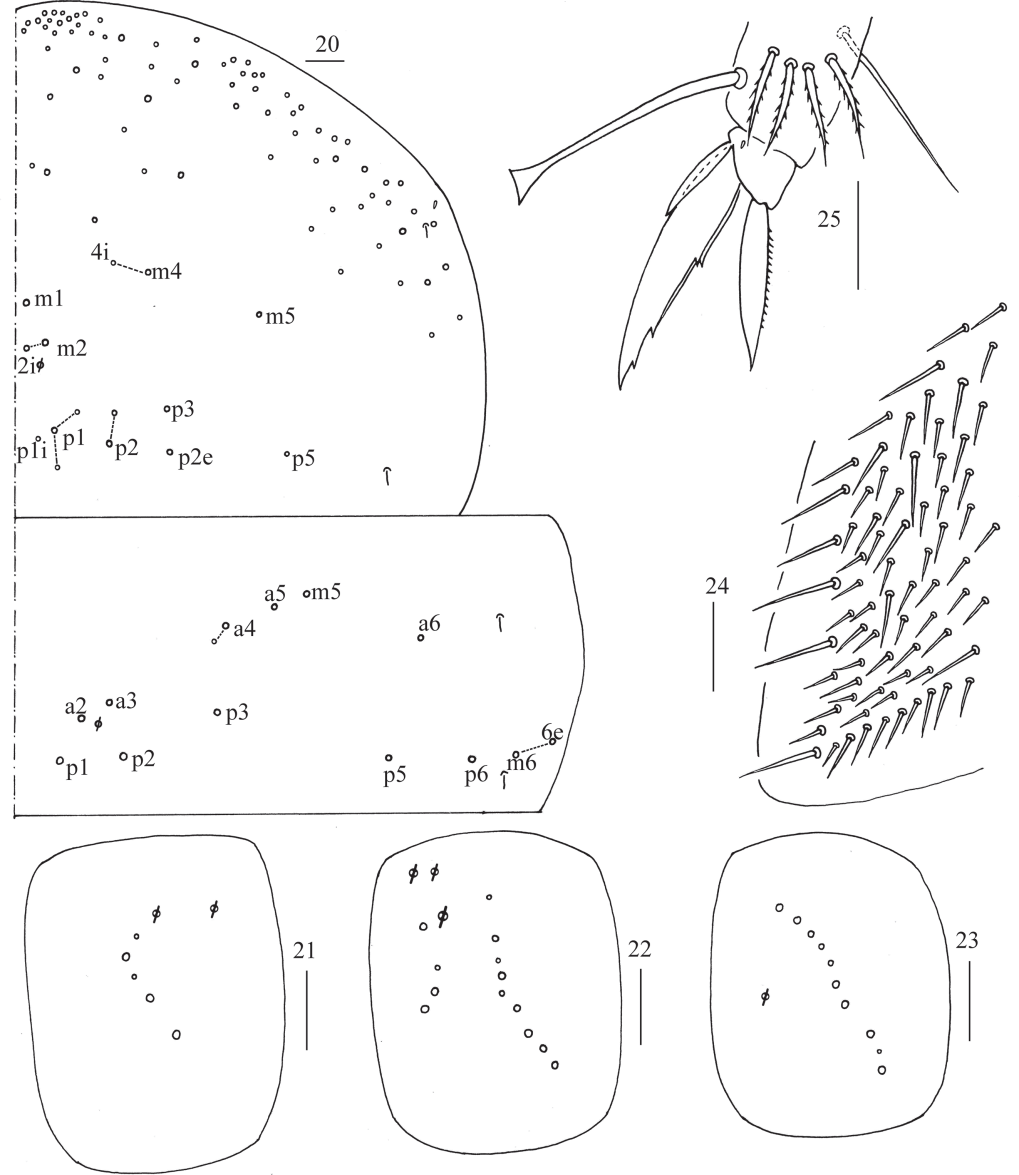
*Lepidodens maculata* sp. nov. **20**. chaetotaxy of Th. II–III (right side); **21–23**. coxal chaetotaxy of fore, middle and hind leg; **24**. trochanteral organ (ventral view); **25**. hind foot complex (lateral view). Scale bars: 20 μm.

***Thorax***: Th. II with three medio-medial (m_1_, m_2_, m_2i_), two medio-sublateral (m_4_, m_4i_), nine posterior mac, one ms and two sens. Th. III with four (a_2_, a_3_, p_1_, p_2_) central and 10 lateral mac, two sens (Fig. 20). Coxal chaetal formula as 5–6/4, 9–11/10 (Figs 21–23). Trochanteral organ with about 73 smooth chaetae (Fig. 24). Tenent hair clavate, 0.96–1.07 length of inner edge of unguis; unguis with four inner teeth, basal pair located at 0.29–0.37 distance from base of inner edge of unguis, distal unpaired teeth at 0.67–0.74 and 0.86–0.90 distance from base; unguiculus lanceolate, outer edge slightly serrate (Fig. 25).

**Figures 26–31.**
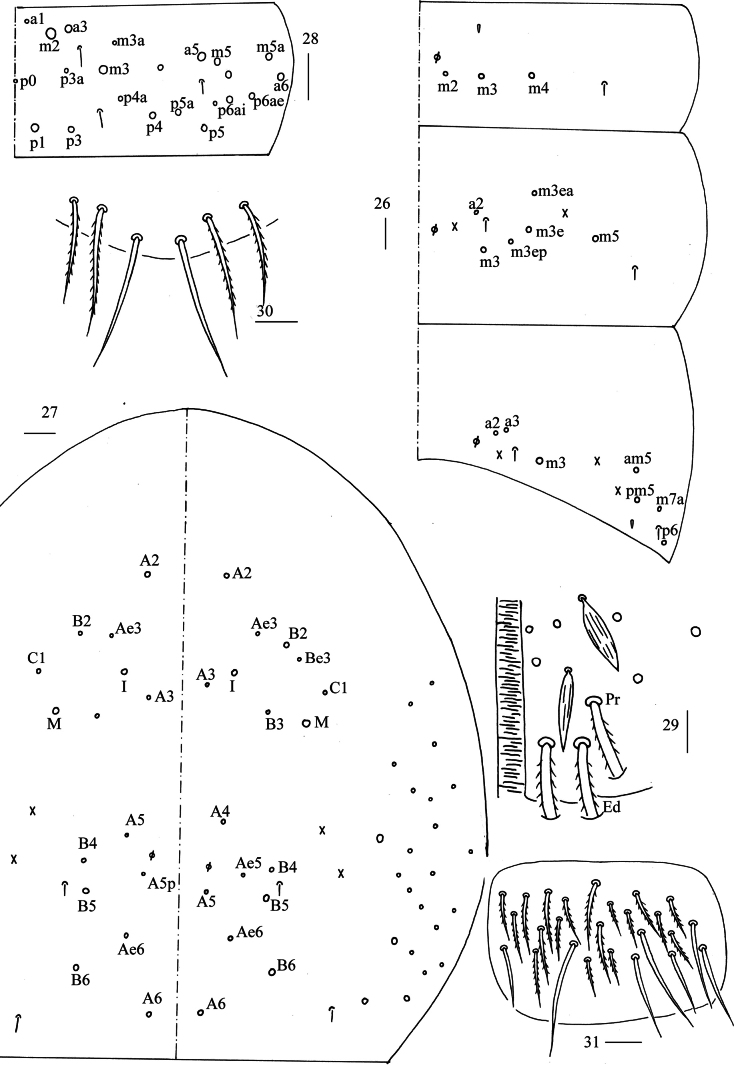
*Lepidodens maculata* sp. nov. **26**. chaetotaxy of Abd. I–III (right side); **27**. chaetotaxy of Abd. IV (right side and left side partially); **28**. chaetotaxy of Abd. V (right side); **29**. anterior face of ventral tube distally (Pr = proximal mac; Ed = external-distal mac); **30**. posterior face of ventral tube apically; **31**. lateral flap of ventral tube. Scale bars: 20 μm.

**Figures 32–34.**
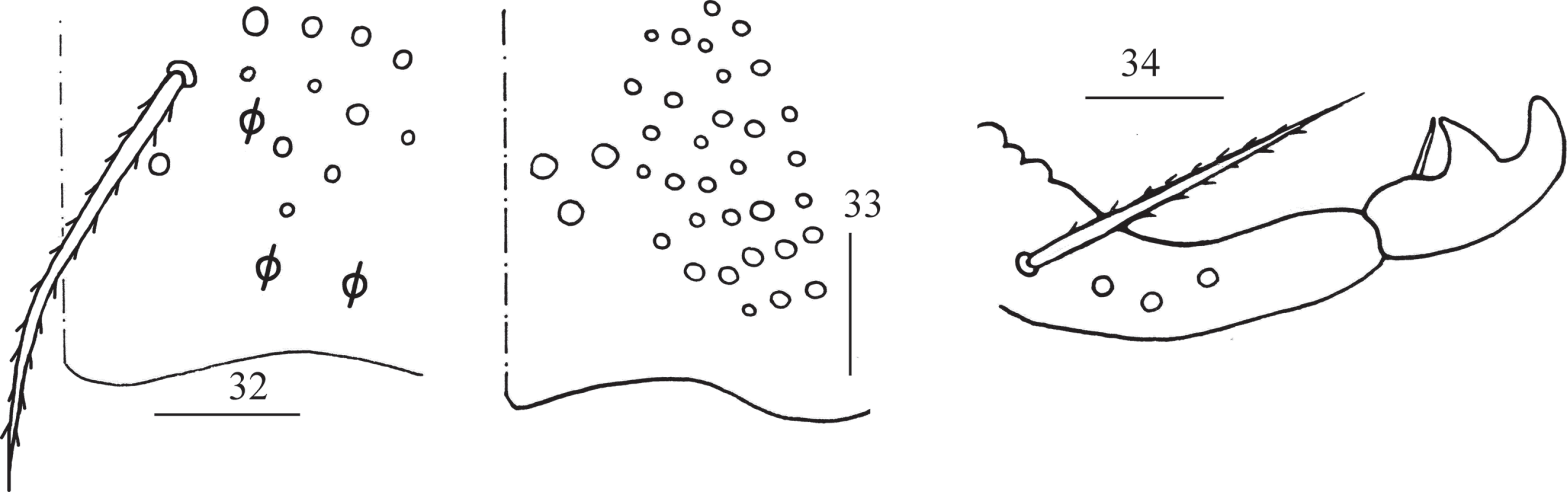
*Lepidodens maculata* sp. nov. **32**. manubrial plaque (dorsal view); **33**. ventro-apical part of manubrium; **34**. mucro (lateral view). Scale bars: 20 μm.

***Abdomen***: Range of Abd. IV length as 5.90–9.00 times dorsal axial length of Abd. III. Tergal ms formula on Abd. I–Abd. V as 1, 0, 1, 0, 0, sens as 1, 2, 2, 2, 3. Abd. I with three (m_2–4_) mac and ms anterior to m_3_. Abd. II with five (a_2_, m_3_, m_3e_, m_3ep_, m_3ea_ rarely absent) central, one (m_5_) lateral mac. Abd. III with three (a_2_, a_3_, m_3_) central, four (am_6_, pm_6_, m_7a_, p_6_) lateral mac (Fig. 26). Abd. IV with two normal sens, 14–17 central and 22–24 (rarely 18) lateral mac (Fig. 27). Abd. V with three sens (Fig. 28). Anterior face of ventral tube scaled with 3+3 large and many small ciliate chaetae, line connecting proximal and external-distal mac oblique to median furrow (Fig. 29); posterior face scaled with two apical smooth chaetae besides numerous ciliate chaetae in different size (Fig. 30); each lateral flap with 5–7 smooth and 10–18 ciliate chaetae (Fig. 31). Manubrial plate dorsally with 9–13 ciliate mac and 3–4 pseudopores (Fig. 32); ventrally with 26–28 (rarely 35) ciliate chaetae (Fig. 33). Mucro bidentate; tip of basal spine reaching apex of subapical tooth; distal smooth section of dens almost equal to mucro in length (Fig. 34).

##### Etymology.

Named after small sublateral blue spots on Th. III and Abd. IV.

##### Ecology.

Found in litter of subtropical forest, mainly composed of leaves of *Castanopsis fargesii*, *Alangium chinensis*, and *Rhododendron simsii*.

##### Remarks.

The new species is very similar to the species *Lepidodens huadingensis* Guo & Pan, 2022 in the chaetotaxy of the dorsal head and abdomen, but there are differences in their colour pattern and the chaetotaxy of the thorax (Table 1). The chaetotaxy on the dorsal body is very useful in the taxonomy of Collembola. We document the chaetotaxy of every specimen of the new species described in the present paper in Table 2.

**Table 1.** Main differences between *L. maculata* sp. nov. and other species of the genus.

Characters	*L. maculata* sp. nov.	*L. huadingensis*	*L. hainanicus*	*L. nigrofasciatus*	*L. similis*	*L. taishunensis*
**Colour pattern on Th. III**	a pair of small blue sublateral spots present	blue pigmented laterally	no blue pigmented	blue pigmented laterally	blue pigmented	no blue pigmented
**Blue pigment on whole Abd. I–III**	no	no	no	yes	yes	no
**Scales on ventral tube**	present	absent	absent	absent	present	absent
**Lateral process of labial papilla E**	reaching or exceeding	not reaching	not reaching	not reaching	not reaching	reaching
**Antennal mac on dorsal head**	7–8	11	8–9	5	5	unknown
**Sutural mac on dorsal head**	6	6	3	4	4	unknown
**Central mac on Th. III**	4	8	3	4	5	6
**Mac on Abd. I**	3	3	1	2	2	2
**Central mac on Abd. II**	5	5	3	3	3	3
**Central mac on Abd. IV**	14–17	19	12–16	6–8	7–13	14

**Table 2.** Intraspecific variation of dorsal chaetotaxy of the new species described in the present paper (? = not clearly seen).

Species	Specimen number	Head				Th. II		Th. III		Abd. I	Central Abd. II	Abd. III		Abd. IV	
		An	interocular chaetae	Gr. II	labial base	Mm	posterior	central	lateral			central	lateral	central	lateral
*Lepidodens maculata* sp. nov.	1281-1A	8	pqvrt	3	M_1_M_2_REL_1_L_2_	3	9	4	10	3	5	3	4	15,16	23,24
	1281-1B	8	pqvrt	3	M_1_M_2_REL_1_L_2_	3	9	4	10	3	5	3	4	15	23
	1307-17	8	pqvrt	3	M_1_M_2_REL_1_L_2_	3	9	4	10	3	5	3	4	16	22
	1307-18	7	pqvrt	3	MREL_1_L_2_	3	9	4	10	3	?	3	4	14	?
	1314-3	8	pqvrt	3	M_1_M_2_REL_1_L_2_	3	9	4	?	3	4,5	3	4	17	?
	1314-4	8,9	pqvrt	3	MREL_1_L_2_	3	9	4	10	3	5	3	4	15,16	18
*Lepidosira cheni* sp. nov.	1274-8	5	pvt	?	M1M_2_REL_1_L_2_	2	10,11	4	9	2	4	2,3	5	8,9	17
	1279-1	6	pvt	2	MREL_1_L_2_	2	10,11	4	9	2	3	3	5	8,9	17
	1279-8A	6	pvt	2	MREL_1_L _2_	2	10,11	4	9	2	3	3	5	8	16
	1279-8B	6	pvt	2	M_1_M_2_REL_1_L_2_	2	11	4	?	2	3	3	5	9	21
*Lepidosira guilinensis* sp. nov.	1274-11	8	pvt	1	M_1_M_2_M_3_REL_1_L_2_	2	15	5	11	3	4	2	4	16	19
	1274-14	7	pvt	1	M_1_M_2_M_3_REL_1_L_2_	2	14	5	11	3	4	2	4	15,16	20
	1277-9	8	pvt	1	M_1_M_2_M_3_REL_1_L_2_	2	15	5	11	3	4	2	4	14,16	19
*Lepidosira montis* sp. nov.	1321-22	11	pvt	1	M_1_M_2_M_3_REL_1_L_2_	1	17	8	10	4	5	2	4	17,19	20,21
	1321-24	10,11	pvt	1	M_1_M_2_M_3_REL_1_L_2_	1	16	8	10	4	5	2	4	18,19	19
*Willowsia zhangi* sp. nov.	1310-7A	5,6	pvt	?	M_1_M_2_REL_1_L_2_	2	17	8	14	3	4	3	5	8	19
	1310-7B	5,6	pvt	3	M_1_M_2_REL_1_L_2_	2	19	8	14	3	4	3	5	8	18,19
	1310-7C	6	pvt	3	M_1_M_2_REL_1_L_2_	2	16,20	8	14	3,4	4	3	5	8,9	16,21
	1310-7D	6,7	pvt	3	M_1_M_2_REL_1_L_2_	2	18	8	14	3	4	3	5	8	20

#### Genus Lepidosira

Taxon classificationAnimaliaEntomobryomorphaEntomobryidae

Schött, 1925

9C6A0E50-72E1-5022-B895-8C74CF9340CD

##### Type species.

*Lepidosira montana* Schött, 1925: 116.

#### Lepidosira cheni sp. nov.

Taxon classificationAnimaliaEntomobryomorphaEntomobryidae

E8D1C3CC-1362-5E4A-B575-7C4CA4CFCBAA

https://zoobank.org/9D2AE746-1785-470D-A7BE-39EACA01E317

Figs 35, 36, 37, 38, 39, 40, 41, 42, 43, 44, 45, 46, 47, 48, 49, 50, 51, 52, 53, 54, 55, 56, 57, 58, 59, 60, 61, 62, 63, 64, 65, Tables 2, 3

##### Type material.

***Holotype*** • ♀ on slide, China, Guangxi Autonomous Region, Guilin City, Longsheng Autonomous County, Huaping Natural Reserve, Anjiangping Protection Station, 26-V-2023, 25°33'44"N, 109°56'16"E, 1341.0 m asl, sample number 1274. ***Paratypes*** • 3♀♀ on slides, China, Guangxi Autonomous Region, Guilin City, Longsheng Autonomous County, Huaping Natural Reserve, Yunxigu Scenic Spot, 29-V-2023, 25°33'25"N, 109°56'39"E, 1340.5 m asl, sample number 1279. All collected by Y-T Ma.

##### Description.

***Size***: Body length up to 2.15 mm.

**Figure 35.**
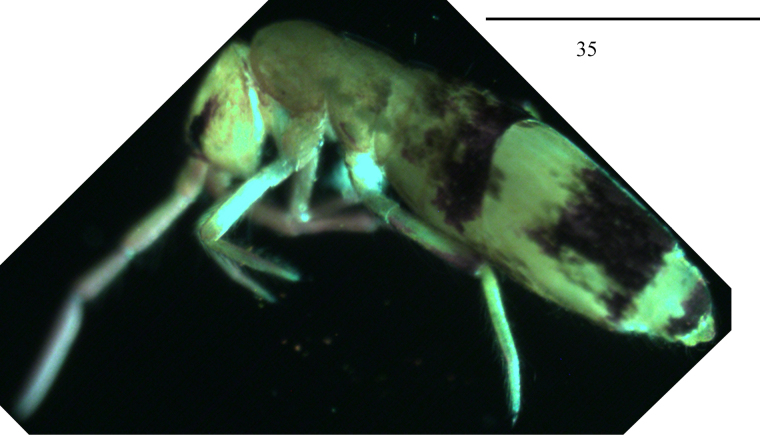
Habitus of *Lepidosira cheni* sp. nov. (lateral view). Scale bar: 1 mm.

***Colour pattern***: Ground colour pale yellow. Eye patches dark blue; Abd. III blue pigmented almost entirely; posterior parts of Abd. IV and Abd. V also blue pigmented; distal parts of antenna and femur of hind leg with scattered blue pigment (Fig. 35).

**Figures 36–42.**
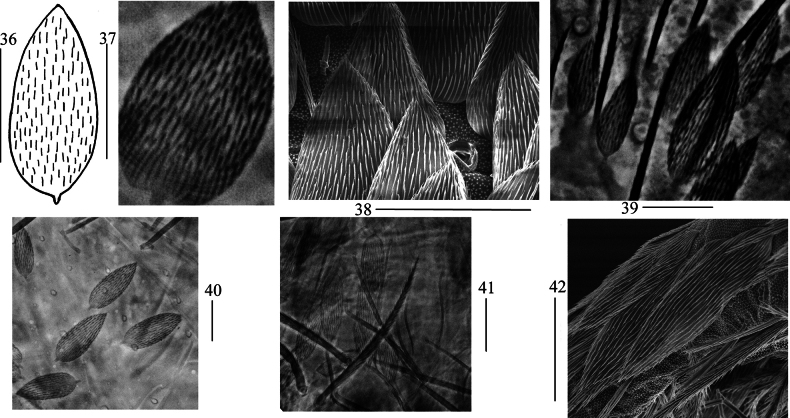
Scales of *Lepidodens cheni* sp. nov. **36**. scale (dorsal view); **37**. photomicrograph of scale (dorsal view); **38**. SEM photomicrograph of scales on terga (dorsal view); **39**. photomicrograph of scales on Ant. I–II (dorsal view); **40**. photomicrograph of scales on head (dorsal view); **41**. photomicrograph of scales on ventral tube (anterior view); **42**. SEM photomicrograph of scales on ventral dens. Scale bars: 20 μm.

***Scale***: Scales spinulated type (Figs 36, 37), present on terga (Fig. 38), Ant. I–II (Fig. 39), head (Fig. 40), legs, ventral tube (Fig. 41), ventral side of manubrium and dentes (Fig. 42).

**Figures 43–50.**
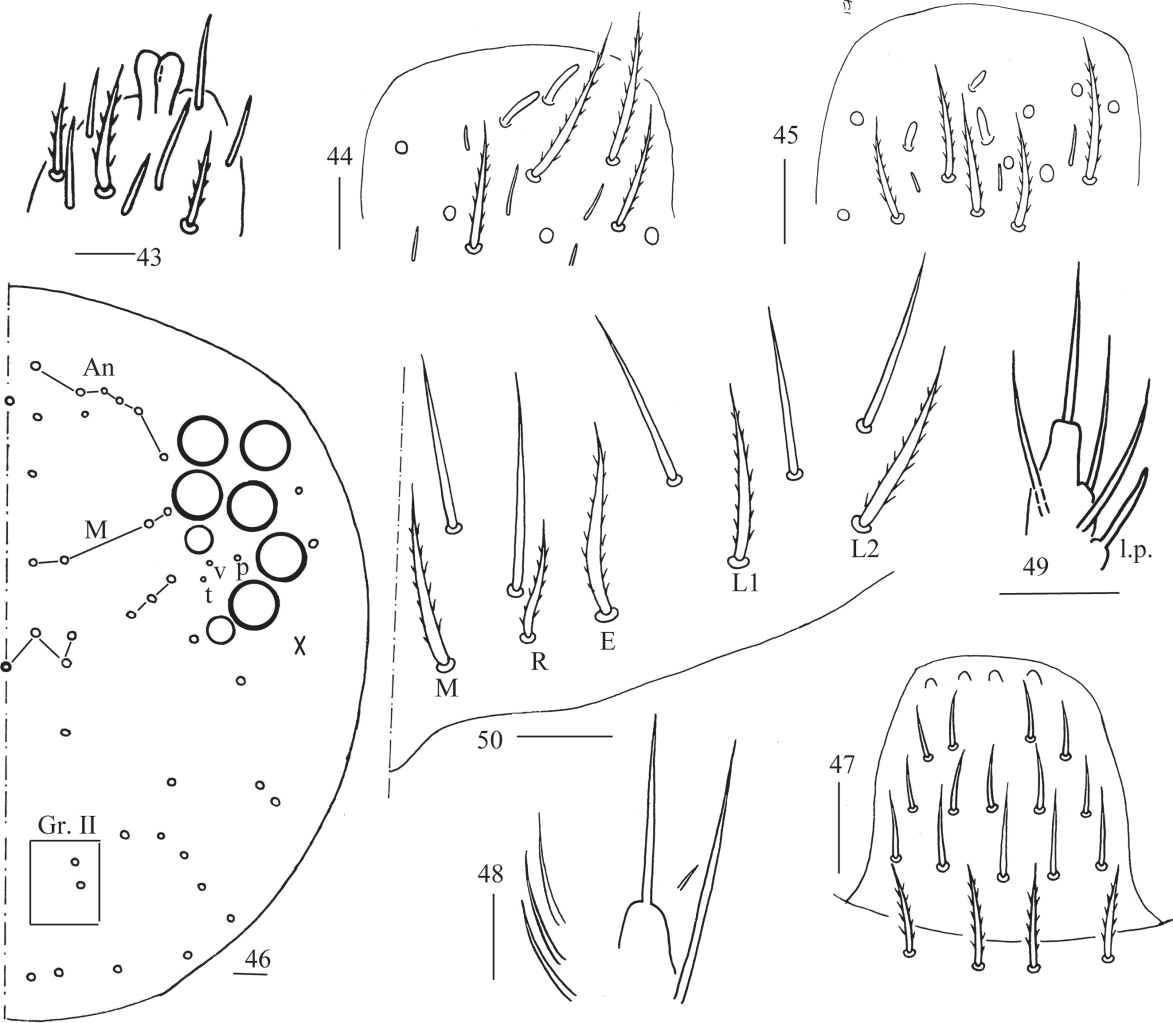
*Lepidosira cheni* sp. nov. **43**. apex of Ant. IV (dorsal view); **44**. distal Ant. III (ventral view); **45**. distal Ant. II (ventral view); **46**. dorsal head (right side); **47**. prelabrum and labrum (dorsal view); **48**. maxillary palp and outer lobe (right side); **49**. labial papilla E (right side); **50**. labial and post-labial chaetotaxy (right side). Scale bars: 20 μm.

***Head***: Antenna not annulated and 0.47–0.57 times length of body. Ratio of Ant. I–IV as 1.00/1.36–1.80/1.30–1.39/2.00–3.00. Distal part of Ant. IV with many sensory chaetae and normal ciliate chaetae, apical bulb bilobed (Fig. 43). Sensory organ of Ant. III with two rod-like chaetae (Fig. 44). Ant. II with two rod-like sensilla apically (Fig. 45). Eyes 8+8, G and H smaller than others, interocular area with p, v, t setae. Dorsal chaetotaxy of head with 5–6 antennal (An), four median (M), seven sutural (S) mac and two mac in Gr. II (Fig. 46). Prelabral and labral chaetae as 4/5, 5, 4, prelabral chaetae ciliate and other smooth, labral papillae round (Fig. 47). Basal chaeta on maxillary outer lobe almost as thick as apical one; sublobal plate with three long and one short smooth chaetae-like processes (Fig. 48). Lateral process of labial papilla E differentiated, with tip not reaching apex of papilla E (Fig. 49). Labial base with MREL_1_L_2_, M_2_ rarely present, all ciliate (Fig. 50).

**Figures 51–56.**
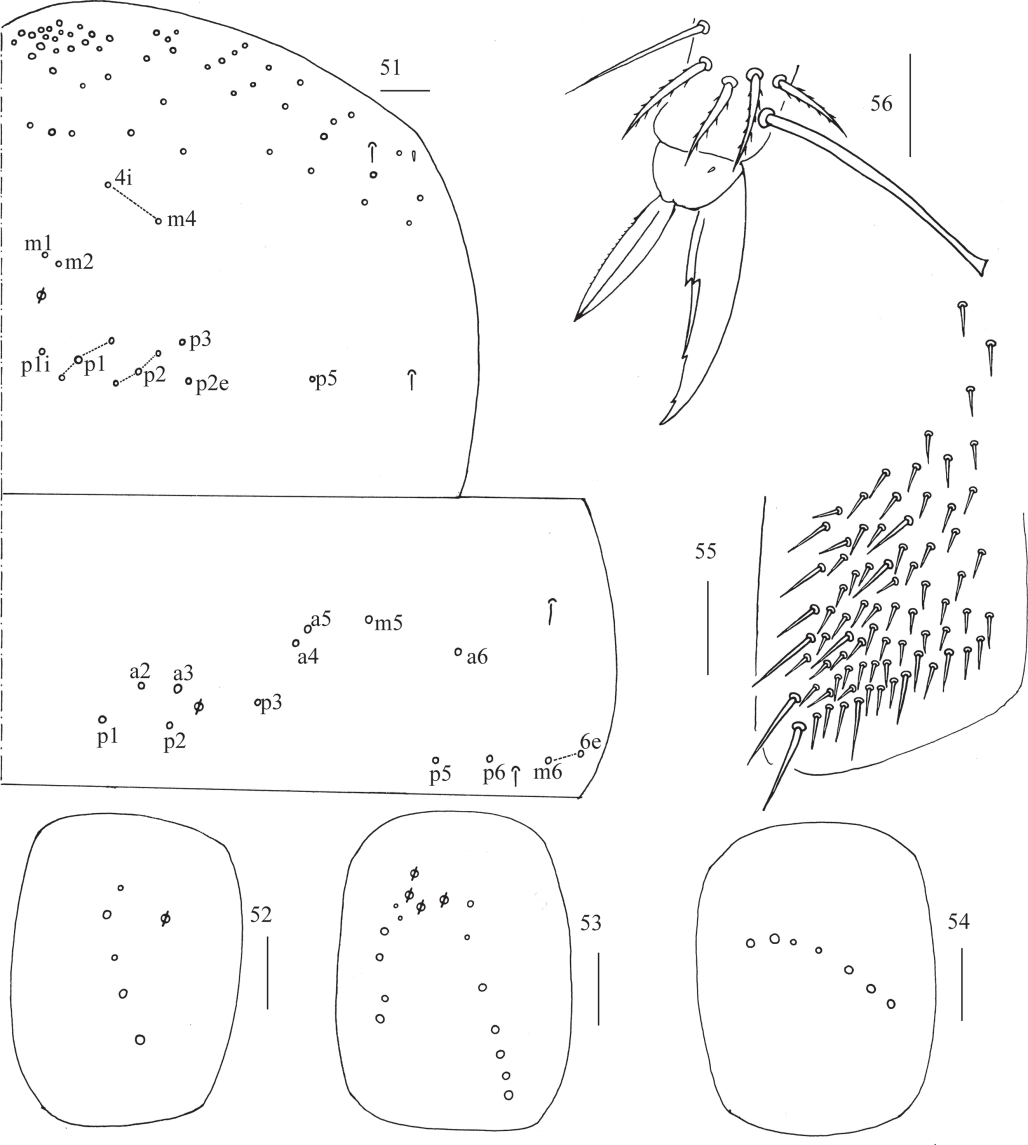
*Lepidosira cheni* sp. nov. **51**. chaetotaxy of Th. II–III (right side); **52–54**. coxal chaetotaxy of fore, middle and hind leg; **55**. trochanteral organ (ventral view); **56**. hind foot complex (lateral view). Scale bars: 20 μm.

***Thorax***: Th. II with two medio-medial (m_1_, m_2_), two medio-sublateral (m_4_, m_4i_), 10–11 posterior mac, one ms and two sens. Th. III with four (a_2_, a_3_, p_1_, p_2_) central and nine lateral mac, two sens (Fig. 51). Coxal chaetal formula as 5/6, 5–7/7 (Figs 52–54). Trochanteral organ with 48–80 smooth chaetae (Fig. 55). Tenent hair clavate, 1.15–1.22 length of inner edge of unguis; unguis with four inner teeth, basal pair located at 0.33–0.35 distance from base of inner edge of unguis, distal unpaired teeth at 0.66–0.70 and 0.87–0.89 distance from base, respectively; unguiculus lanceolate, outer edge slightly serrate (Fig. 56).

**Figures 57–59.**
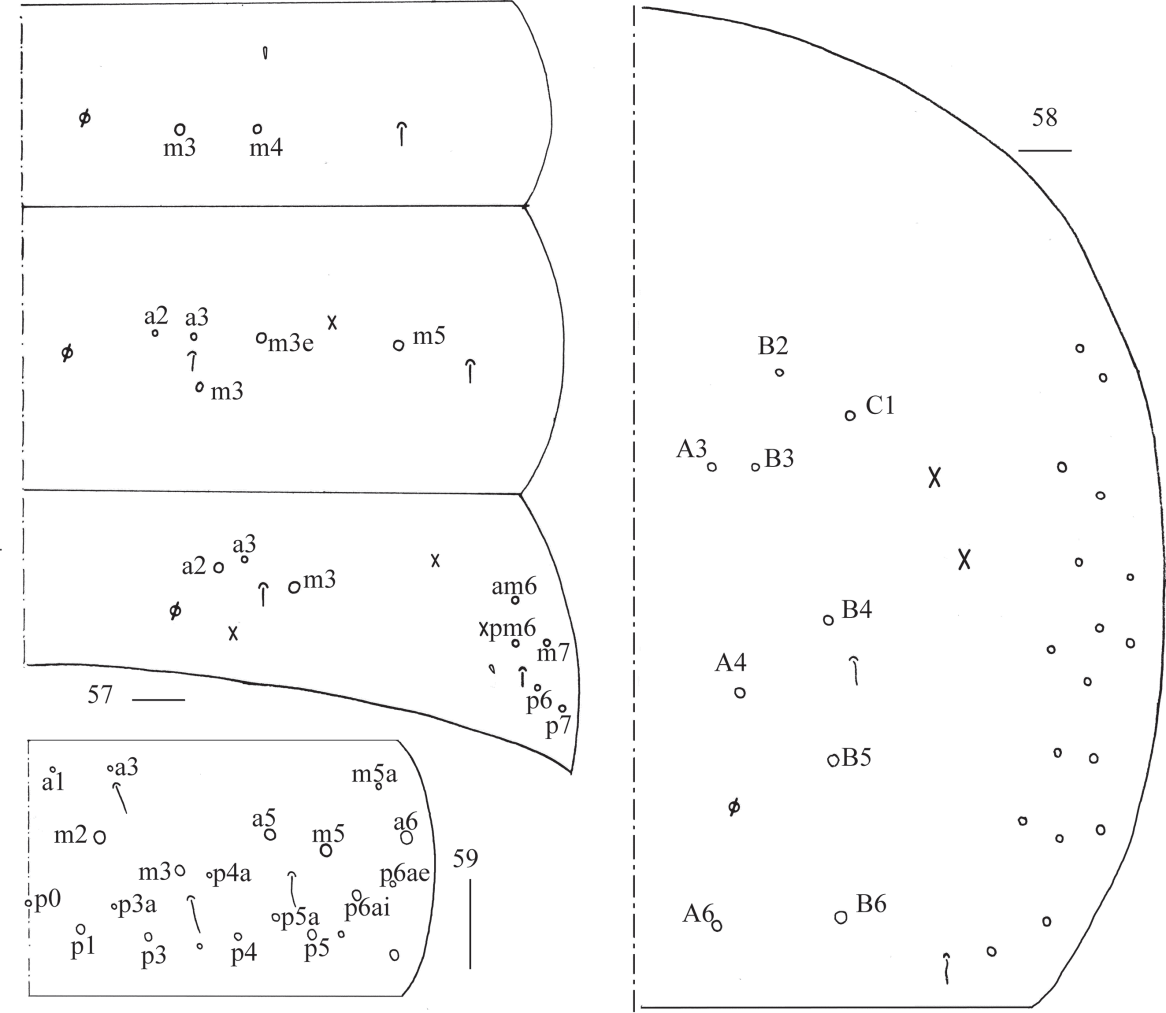
*Lepidosira cheni* sp. nov. **57**. chaetotaxy of Abd. I–III (right side); **58**. chaetotaxy of Abd. IV (right side); **59**. chaetotaxy of Abd. V (right side). Scale bars: 20 μm.

**Figures 60–65.**
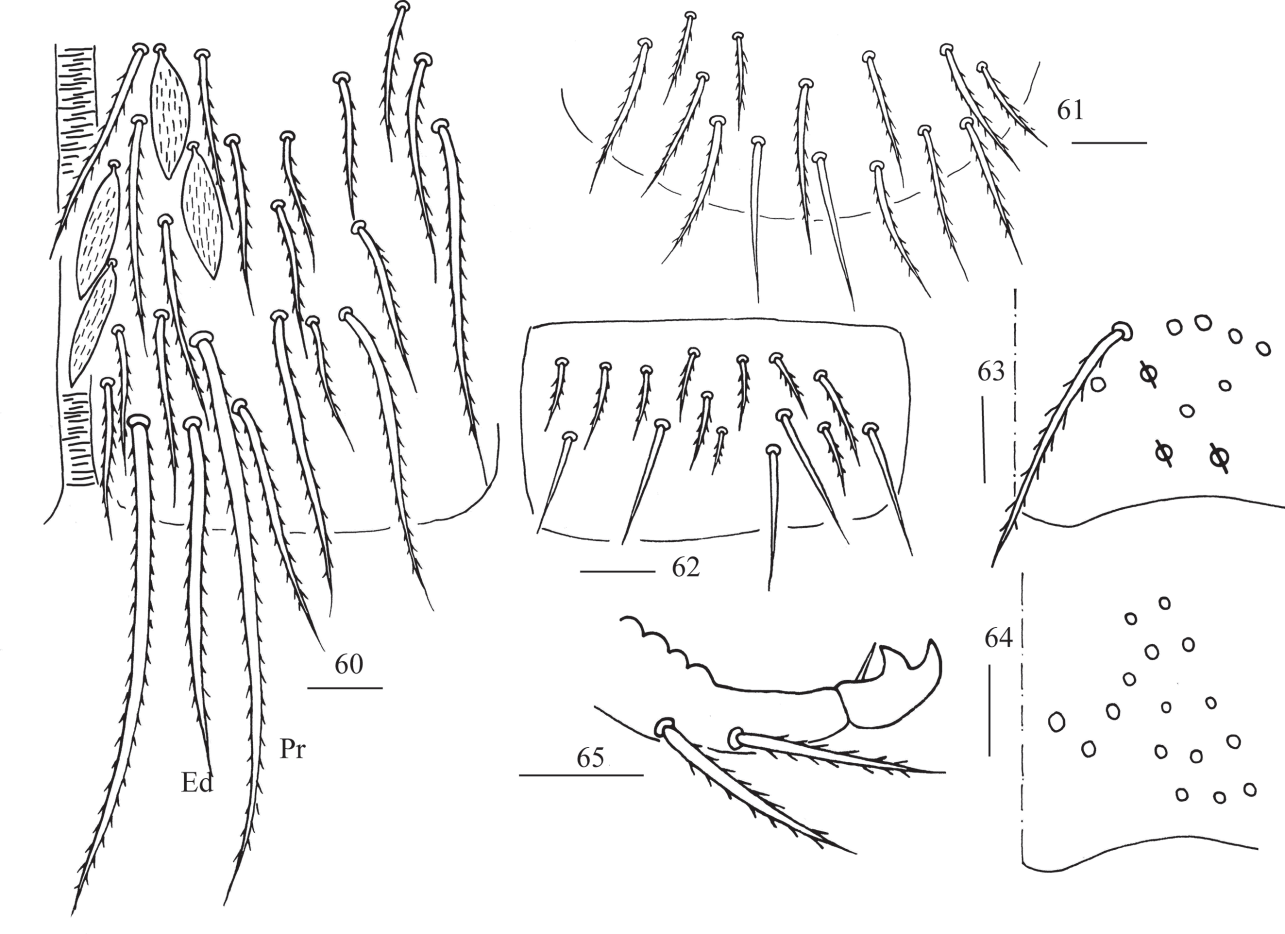
*Lepidosira cheni* sp. nov. **60**. anterior face of ventral tube; **61**. posterior face of ventral tube apically; **62**. lateral flap of ventral tube; **63**. manubrial plaque (dorsal view); **64**. ventro-apical part of manubrium; **65**. mucro (lateral view). Scale bars: 20 μm.

***Abdomen***: Range of Abd. IV length as 4.67–5.70 times dorsal axial length of Abd. III. Tergal ms formula on Abd. I–Abd. V as 1, 0, 1, 0, 0, sens as 1, 2, 2, 2, 3. Abd. I with two (m_3_, m_4_) mac and ms anterior to m_4_. Abd. II usually with 3 (4) (a_2_, m_3_, m_3e_, a_3_ rarely present) central, one (m_5_) lateral mac. Abd. III with three (a_2_, a_3_, m_3_) central, five (am_6_, pm_6_, m_7a_, p_6_, p_7_) lateral mac (Fig. 57). Abd. IV with two normal sens, 8–9 central and 16–21 (rarely 18) lateral mac (Fig. 58). Abd. V with three sens (Fig. 59). Anterior face of ventral tube scaled with 3+3 large and many small ciliate chaetae, line connecting proximal and external-distal mac oblique to median furrow (Fig. 60); posterior face scaled with two apical smooth chaetae besides numerous ciliate chaetae in different size (Fig. 61); each lateral flap with 5–6 smooth and 10 ciliate chaetae (Fig. 62). Manubrial plate dorsally with 5–11 ciliate mac and 3 (rarely 2) pseudopores (Fig. 63); ventrally with 16–20 ciliate chaetae (Fig. 64). Mucro bidentate; tip of basal spine reaching apex of subapical tooth; distal smooth section of dens almost equal to mucro in length (Fig. 65).

##### Etymology.

Named after Prof. Zhilin Chen from Guangxi Normal University, China, who helped to collect the specimens of the new species.

##### Ecology.

Found in litter of subtropical forest, mainly composed of leaves of *Castanopsis chinensis*, *Alangium chinensis*, and *Schima superba*.

##### Remarks.

The new species is characterised by its colour pattern and the position of ms on Abd. I. It is not similar to any known species of *Lepidosira* in colour pattern, and we compare it with the Vietnamese species *L. alba* (Nguyen, 2005) and *L. nigropunctata* (Nguyen, 2005) and the Indian species *L. unguserrata* Salmon, 1970. Their differences are listed in Table 3.

**Table 3.** Main differences between *Lepidosira cheni* sp. nov. and similar species.

Characters	*L. cheni* sp. nov.	*L. alba*	*L. nigropunctata*	*L. unguserrata*
A blue stripe on Abd. III	present	absent	absent	absent
A rectangular blue patch on Abd. IV	present	absent	absent	absent
Central mac on Th. III	4	5	10	unknown
Mac on Abd. I	2	2	8	unknown
Central mac on Abd. II	4	5	5	unknown

#### Lepidosira guilinensis sp. nov.

Taxon classificationAnimaliaEntomobryomorphaEntomobryidae

3F873054-CE3F-5B3C-94B3-8DF76967105B

https://zoobank.org/7F916622-C7D8-4DD6-B2B6-8CDC6880B27A

Figs 66, 67, 68, 69, 70, 71, 72, 73, 74, 75, 76, 77, 78, 79, 80, 81, 82, 83, 84, 85, 86, 87, 88, 89, 90, 91, 92, 93, 94, 95, 96, 97, 98, 99, Tables 2, 4

##### Type material.

***Holotype*** • ♀ on slide, China, Guangxi Autonomous Region, Guilin City, Longsheng Autonomous County, Huaping Natural Reserve, Guangfu Mountain, 28-V-2023, 25°33'42"N, 109°56'13"E, 1342.8 m asl, sample number 1277. ***Paratypes*** • 2♀♀ on slides, China, Guangxi Autonomous Region, Guilin City, Longsheng Autonomous County, Huaping Natural Reserve, Anjiangping Protection Station, 26-V-2023, 25°33'44"N, 109°56'16"E, 1341.0 m asl, sample number 1274. All collected by Y-T Ma.

**Table 4.** Main differences between *L. guilinensis* sp. nov. and *L. montis* sp. nov. and their similar species.

Characters	*L. guilinensis* sp. nov.	*L. montis* sp. nov.	*L. nigropunctata*	L. terraereginae*
Anmac on head	7–8	10–11	unknown	unknown
Medio-medial mac on Th. II	2	1	2	unknown
Posterior mac on Th. II	14–15	16–17	15	3
Central mac on Th. II	5	8	10	0
Mac on Abd. I	3	4	8	0
Central mac on Abd. II	4	5	5	3
Central mac on Abd. III	2	2	2	1
Inner teeth on unguis	3	4	3	2
Ventral tube	scaled	scaled	unscaled	unscaled

*Based on Yoshii and Greenslade’s description (1994).

##### Description.

***Size***: Body length up to 2.39 mm.

**Figures 66–68.**
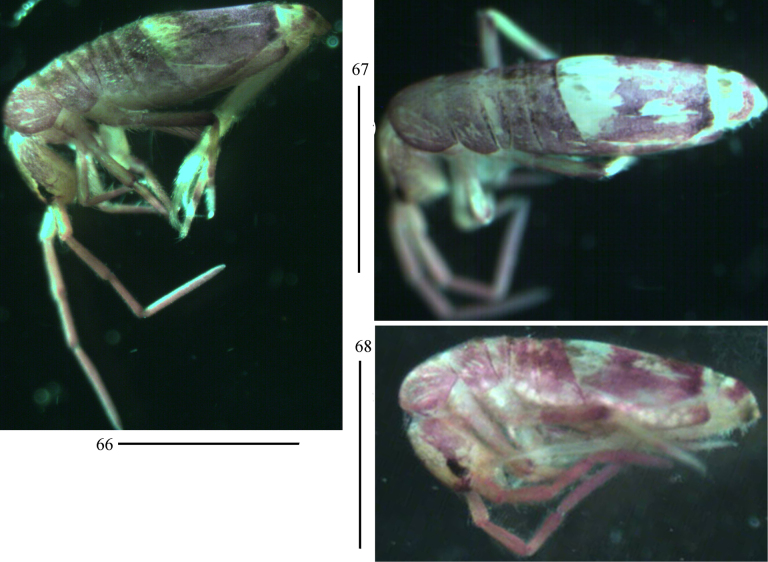
Habitus of *Lepidosira guilinensis* sp. nov. (lateral view). Scale bar: 1 mm.

***Colour pattern***: Ground colour pale yellow. Eye patches dark blue; Th. II–Abd. III brown pigmented; antenna, lateral part of dorsal head, legs, posterior parts of Abd. IV and Abd. V also with brown pigment (Figs 66–68).

***Scales***: Scales spinulated type (Fig. 69), present on terga (Fig. 70), Ant. I–II (Fig. 71), head, legs (Fig. 72), ventral tube (Fig. 73), ventral side of manubrium and dentes (Figs 74–76).

**Figures 69–76.**
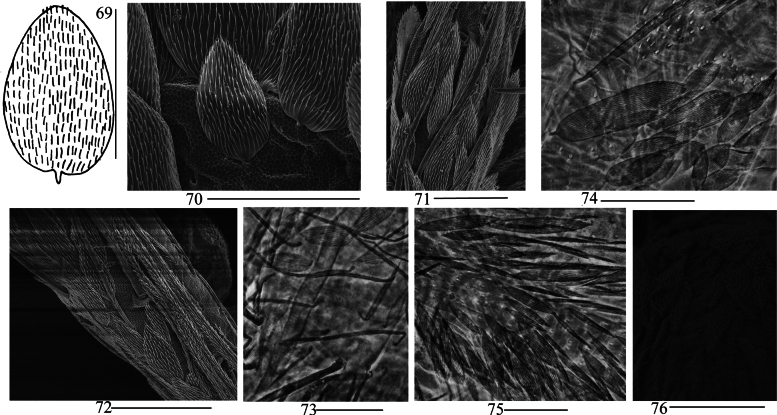
Scales of *Lepidosira guilinensis* sp. nov. **69**. scale (dorsal view); **70**. SEM photomicrograph of scales on terga (dorsal view); **71**. SEM photomicrograph of scales on Ant. I–II (dorsal view); **72**. SEM photomicrograph of scales on leg; **73**. photomicrograph of scales on ventral tube (anterior view); **74**. photomicrograph of scales on ventral manubrium; **75**. photomicrograph of scales on ventral dens; **76**. SEM photomicrograph of scales on ventral dens. Scale bars: 20 μm.

**Figures 77–84.**
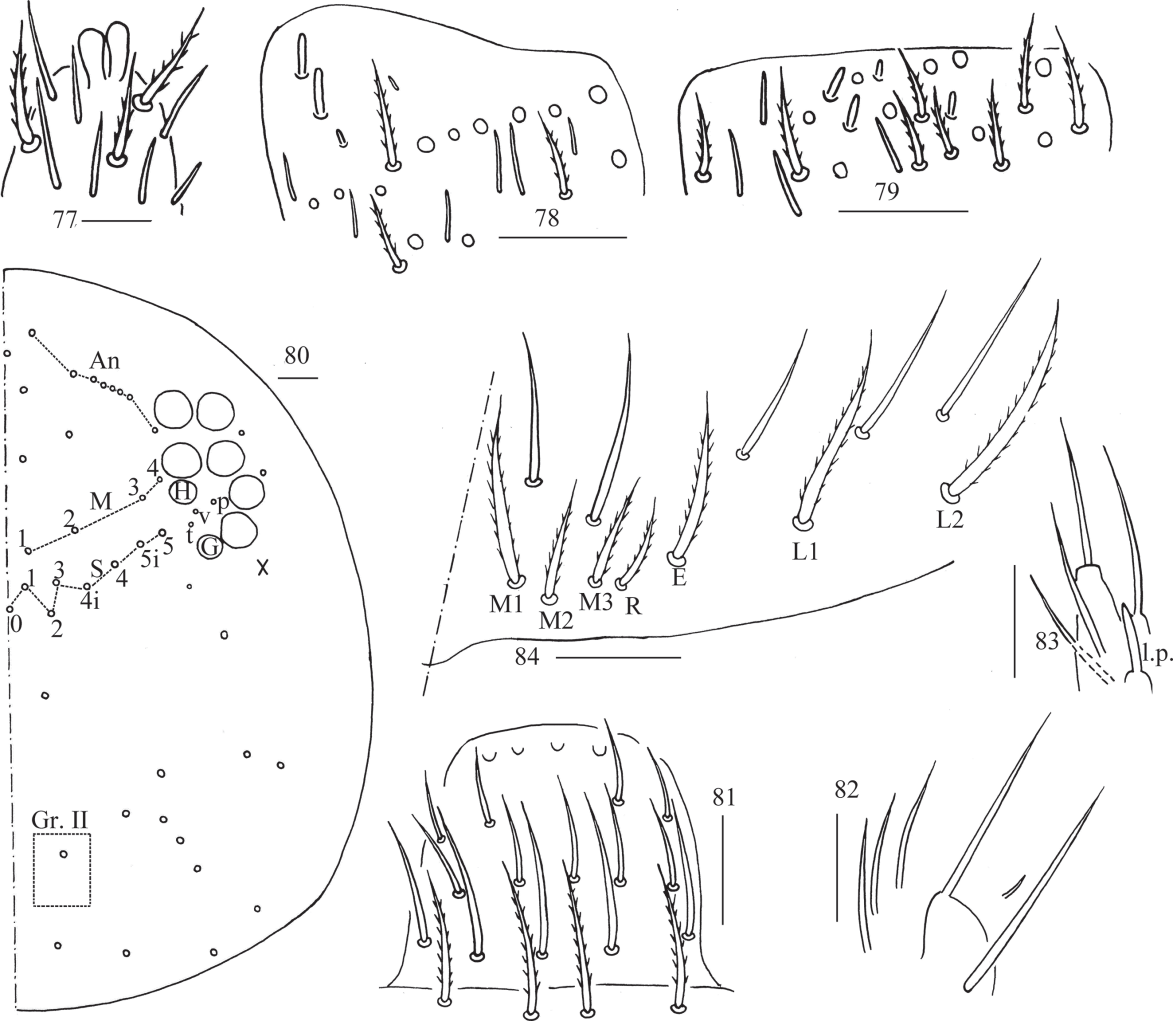
*Lepidosira guilinensis* sp. nov. **77**. apex of Ant. IV (dorsal view); **78**. distal Ant. III (ventral view); **79**. distal Ant. II (ventral view); **80**. dorsal head (right side); **81**. prelabrum and labrum (dorsal view); **82**. maxillary palp and outer lobe (right side); **83**. labial papilla E (right side); **84**. labial and post-labial chaetotaxy (right side). Scale bars: 20 μm.

***Head***: Antenna not annulated and 0.49–0.59 times length of body. Ratio of Ant. I–IV as 1.00/1.30–1.73/1.20–1.64/2.30–2.75. Distal part of Ant. IV with many sensory chaetae and normal ciliate chaetae, apical bulb bilobed (Fig. 77). Sensory organ of Ant. III with two rod-like chaetae (Fig. 78). Ant. II with 3–4 rod-like sensilla apically (Fig. 79). Eyes 8+8, G and H smaller than others, interocular interocular area with p, v, t setae. Dorsal chaetotaxy of head with seven or eight antennal (An), four median (M), eight sutural (S) mac and one mac in Gr. II (Fig. 80). Prelabral and labral chaetae as 4/5, 5, 4, prelabral chaetae ciliate and others smooth, labral papillae round (Fig. 81). Basal chaeta on maxillary outer lobe almost as thick as apical one; sublobal plate with three long and one short smooth chaetae-like processes (Fig. 82). Lateral process of labial papilla E differentiated, with tip not reaching apex of papilla E (Fig. 83). Labial base with M_1_M_2_M_3_REL_1_L_2_, all ciliate (Fig. 84).

**Figures 85–90.**
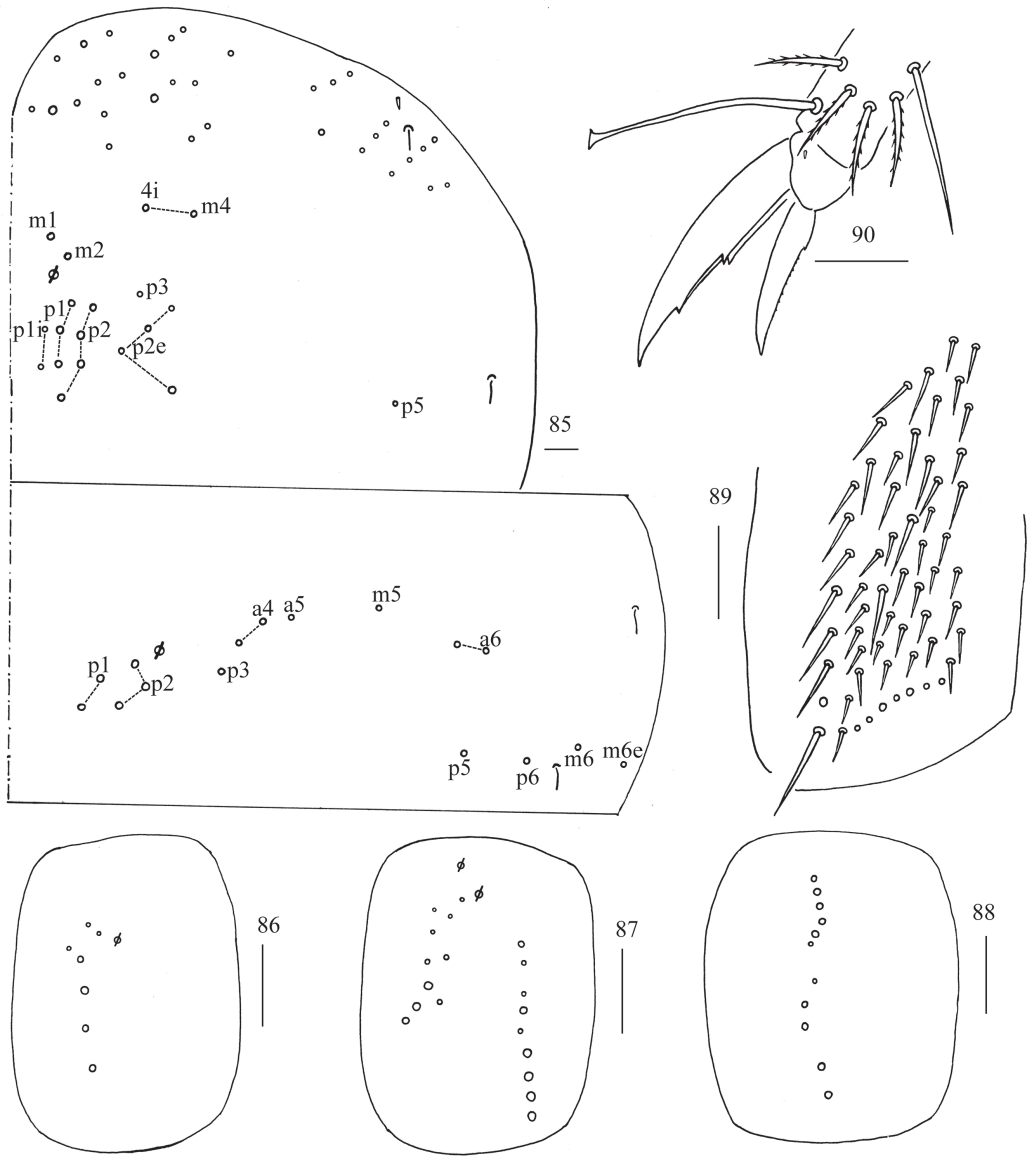
*Lepidosira guilinensis* sp. nov. **85**. chaetotaxy of Th. II–III (right side); **86–88**. coxal chaetotaxy of fore, middle and hind leg; **89**. trochanteral organ (ventral view); **90**. hind foot complex (lateral view). Scale bars: 20 μm.

***Thorax***: Th. II with two medio-medial (m_1_, m_2_), two medio-sublateral (m_4_, m_4i_), 14–15 posterior mac, one ms and two sens. Th. III with five central and 11 lateral mac, two sens (Fig. 85). Coxal chaetal formula as 7/10–11, 9–12/11 (Figs 86–88). Trochanteral organ with 42–63 smooth chaetae (Fig. 89). Tenent hair clavate, 1.01–1.10 length of inner edge of unguis; unguis with three inner teeth, basal pair located at 0.34–0.37 distance from base of inner edge of unguis, distal one at 0.66–0.70 distance from base; unguiculus lanceolate, outer edge slightly serrate (Fig. 90).

**Figures 91–93.**
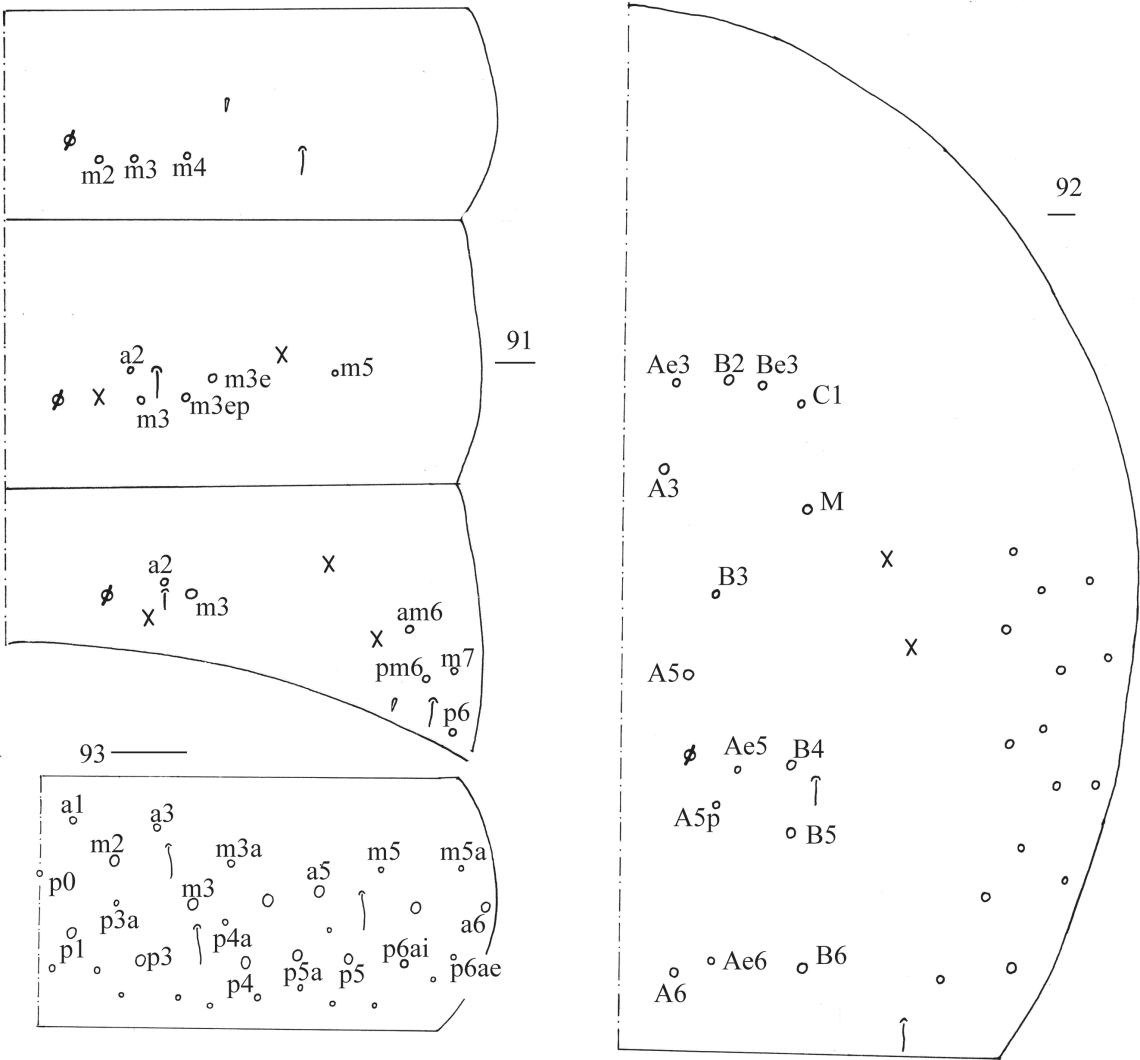
*Lepidosira guilinensis* sp. nov. **91**. chaetotaxy of Abd. I–III (right side); **92**. chaetotaxy of Abd. IV (right side); **93**. chaetotaxy of Abd. V (right side). Scale bars: 20 μm.

**Figures 94–99.**
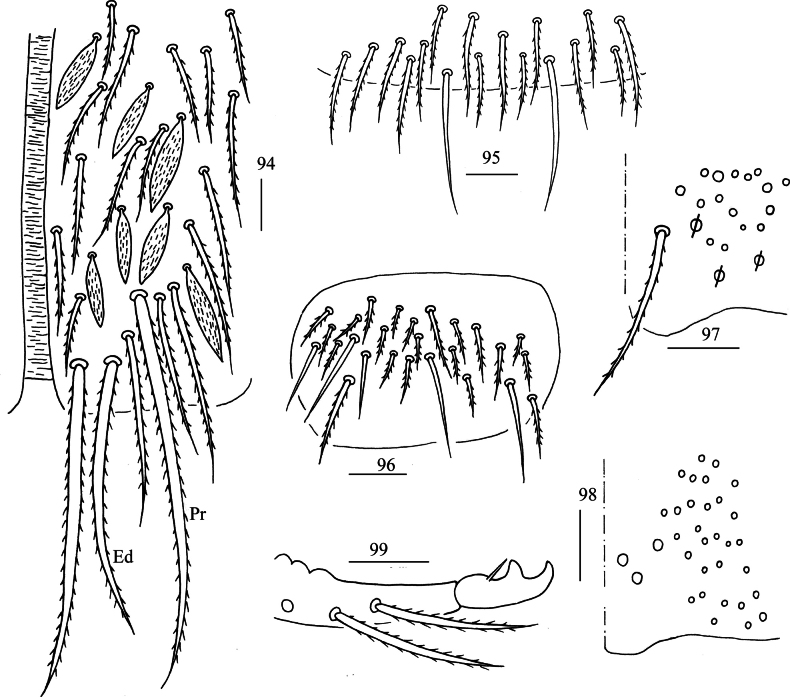
*Lepidosira guilinensis* sp. nov. **94**. anterior face of ventral tube; **95**. posterior face of ventral tube apically; **96**. lateral flap of ventral tube; **97**. manubrial plaque (dorsal view); **98**. ventro-apical part of manubrium; **99**. mucro (lateral view). Scale bars: 20 μm.

***Abdomen***: Range of Abd. IV length as 5.71–6.10 times dorsal axial length of Abd. III. Tergal ms formula on Abd. I–Abd. V as 1, 0, 1, 0, 0, sens as 1, 2, 2, 2, 3. Abd. I with three (m_2_, m_3_, m_4_) mac, ms outer to m4. Abd. II with four (a_2_, m_3_, m_3e_, m_3ep_) central, one (m5) lateral mac. Abd. III with two (a_2_, m_3_) central, four (am_6_, pm_6_, m_7a_, p_6_) lateral mac (Fig. 91). Abd. IV with two normal sens, 14–16 central and 19–20 lateral mac (Fig. 92). Abd. V with three sens (Fig. 93). Anterior face of ventral tube scaled with 3+3 large and many small ciliate chaetae, line connecting proximal and external-distal mac oblique to median furrow (Fig. 94); posterior face scaled with two apical smooth chaetae besides numerous ciliate chaetae in different size (Fig. 95); each lateral flap with five smooth and 16–21 ciliate chaetae (Fig. 96). Manubrial plate dorsally with 17–21 (rarely 10) ciliate mac and three (rarely 2) pseudopores (Fig. 97); ventrally with 27–33 ciliate chaetae (Fig. 98). Mucro bidentate; tip of basal spine reaching apex of subapical tooth; distal smooth section of dens almost equal to mucro in length (Fig. 99).

##### Etymology.

Named after its locality: Guilin City.

##### Ecology.

Found in litter of subtropical forest, mainly composed of leaves of *Castanopsis chinensis*, *Alangium chinensis*, *Schima superba* and *Mallotus tenuifolius*.

#### Lepidosira montis sp. nov.

Taxon classificationAnimaliaEntomobryomorphaEntomobryidae

98FA6D74-DB3D-57EC-B72A-5B00F32691A9

https://zoobank.org/66BB0541-7D41-4D54-8CC7-D345FEED361A

Figs 100, 101, 102, 103, 104, 105, 106, 107, 108, 109, 110, 111, 112, 113, 114, 115, 116, 117, 118, 119, 120, 121, 122, 123, 124, 125, 126, 127, 128, 129, Tables 2, 4

##### Type material.

***Holotype*** • ♀ on slide, China, Chongqing Municipality, Wuxi County, Yintiaoling National Nature Reserve, Guanshan Protection Station, Stone Pillar, 28-VII-2024, 31°32'15"N, 109°41'53"E, 2168.9 m asl, sample number 1321. ***Paratypes*** • ♀ on slide, same data as holotype. All collected by Y-T Ma.

##### Description.

***Size***: Body length up to 3.72 mm.

**Figures 100–101.**
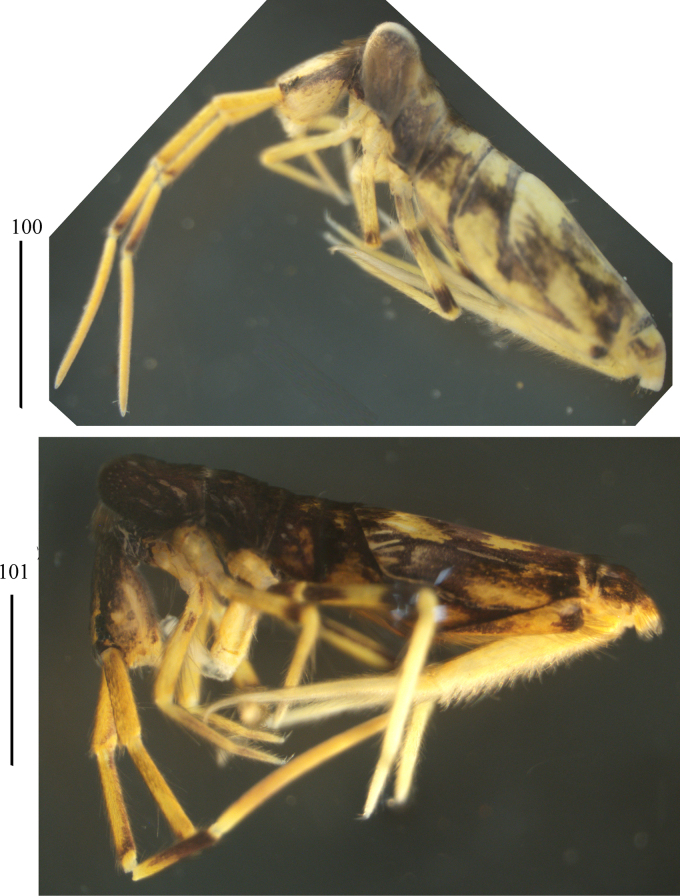
Habitus of *Lepidosira montis* sp. nov. (lateral view). Scale bar: 1 mm.

***Colour pattern***: Ground colour pale yellow. Eye patches dark blue; Th. II–Abd. III brown pigmented almost entirely; Abd. IV and V brown pigmented irregularly; distal Ant. III, lateral part of dorsal head, femur of hind leg also with brown pigment (Figs 100, 101).

**Figures 102–107.**
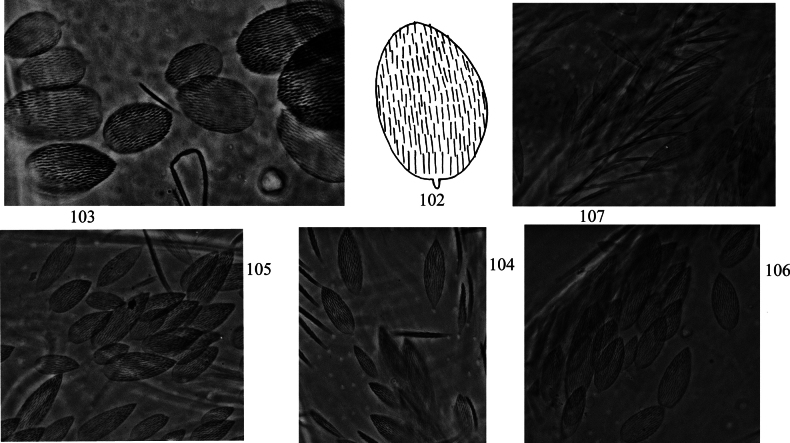
Scales of *Lepidosira montis* sp. nov. **102**. scale (dorsal view); **103**. photomicrograph of scales on terga (dorsal view); **104**. photomicrograph of scales on Ant. I–II (dorsal view); **105**. photomicrograph of scales on dorsal head; **106**. photomicrograph of scales on leg; **107**. photomicrograph of scales on ventral dentes. Scale bars: 20 μm.

***Scales***: Scales spinulated type (Fig. 102), present on terga (Fig. 103), Ant. I–II (Fig. 104), head (Fig. 105), legs (Fig. 106), ventral tube, ventral side of manubrium and dentes (Fig. 107).

**Figures 108–115.**
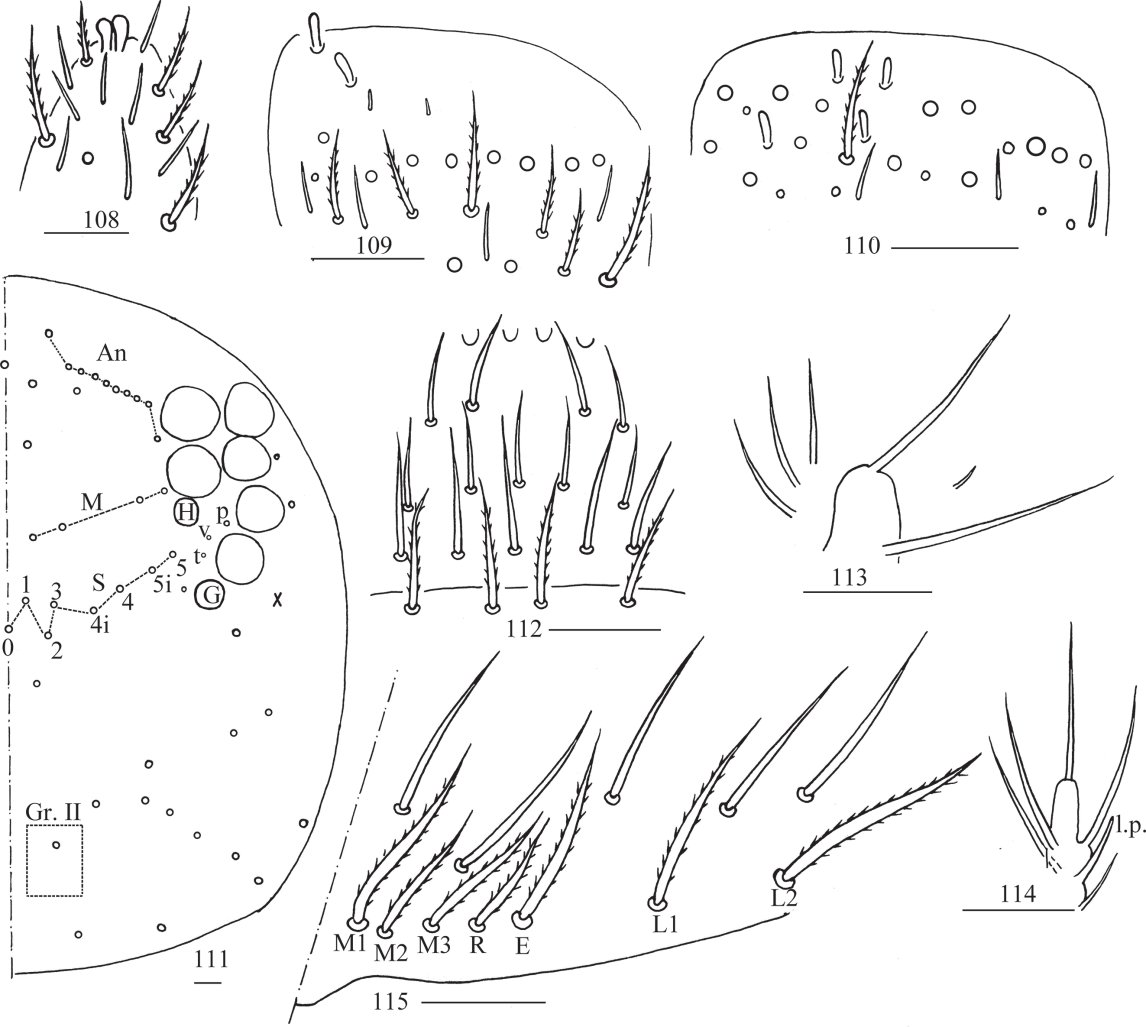
*Lepidosira montis* sp. nov. **108**. apex of Ant. IV (dorsal view); **109**. distal Ant. III (ventral view); **110**. distal Ant. II (ventral view); **111**. dorsal head (right side); **112**. prelabrum and labrum (dorsal view); **113**. maxillary palp and outer lobe (right side); **114**. labial papilla E (right side); **115**. labial and post-labial chaetotaxy (right side). Scale bars: 20 μm.

***Head***: Antenna not annulated and 0.70–0.74 times length of body. Ratio of Ant. I–IV as 1.00/1.14–1.47/0.95–1.23/2.27–2.65. Distal part of Ant. IV with many sensory chaetae and normal ciliate chaetae, apical bulb bilobed (Fig. 108). Sensory organ of Ant. III with two rod-like chaetae (Fig. 109). Ant. II with 2–4 rod-like sensilla apically (Fig. 110). Eyes 8+8, G and H smaller than others, interocular area with p, v, t setae. Dorsal chaetotaxy of head with 10 or 11 antennal (An), four median (M), eight sutural (S) mac and one mac in Gr. II (Fig. 111). Prelabral and labral chaetae as 4/5, 5, 4, prelabral chaetae ciliate and other smooth, labral papillae round (Fig. 112). Basal chaeta on maxillary outer lobe almost as thick as apical one; sublobal plate with three long and one short smooth chaetae-like processes (Fig. 113). Lateral process of labial papilla E differentiated, with tip not reaching apex of papilla E (Fig. 114). Labial base with M_1_M_2_M_3_REL_1_L_2_, all ciliate (Fig. 115).

**Figures 116–120.**
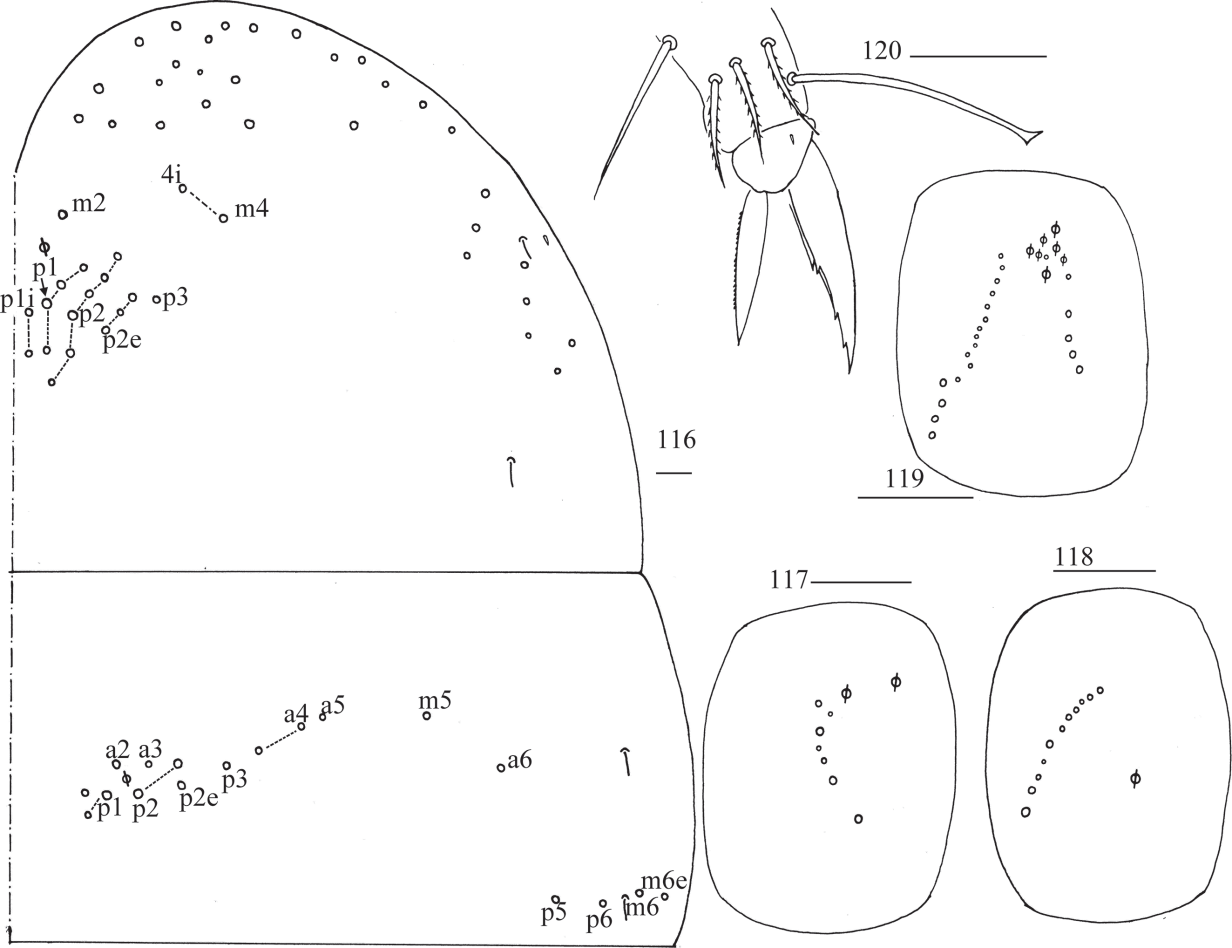
*Lepidosira montis* sp. nov. **116**. chaetotaxy of Th. II–III (right side); **117–119**. coxal chaetotaxy of fore, middle and hind leg; **120**. hind foot complex (lateral view). Scale bars: 20 μm.

***Thorax***: Th. II with one medio-medial (m_2_), two medio-sublateral (m_4_, m_4i_), 16–17 posterior mac, one ms and two sens. Th. III with eight central and 10 lateral mac, two sens (Fig. 116). Coxal chaetal formula as 7/6, 16/11 (Figs 117–119). Trochanteral organ with many smooth chaetae and not clearly seen. Tenent hair clavate, 0.80–1.25 length of inner edge of unguis; unguis with four inner teeth, basal pair located at 0.42–0.48 distance from base of inner edge of unguis, distal teeth at 0.67–0.72 and 0.85–0.89 distance from base, respectively; unguiculus lanceolate, outer edge slightly serrate (Fig. 120).

**Figures 121–126.**
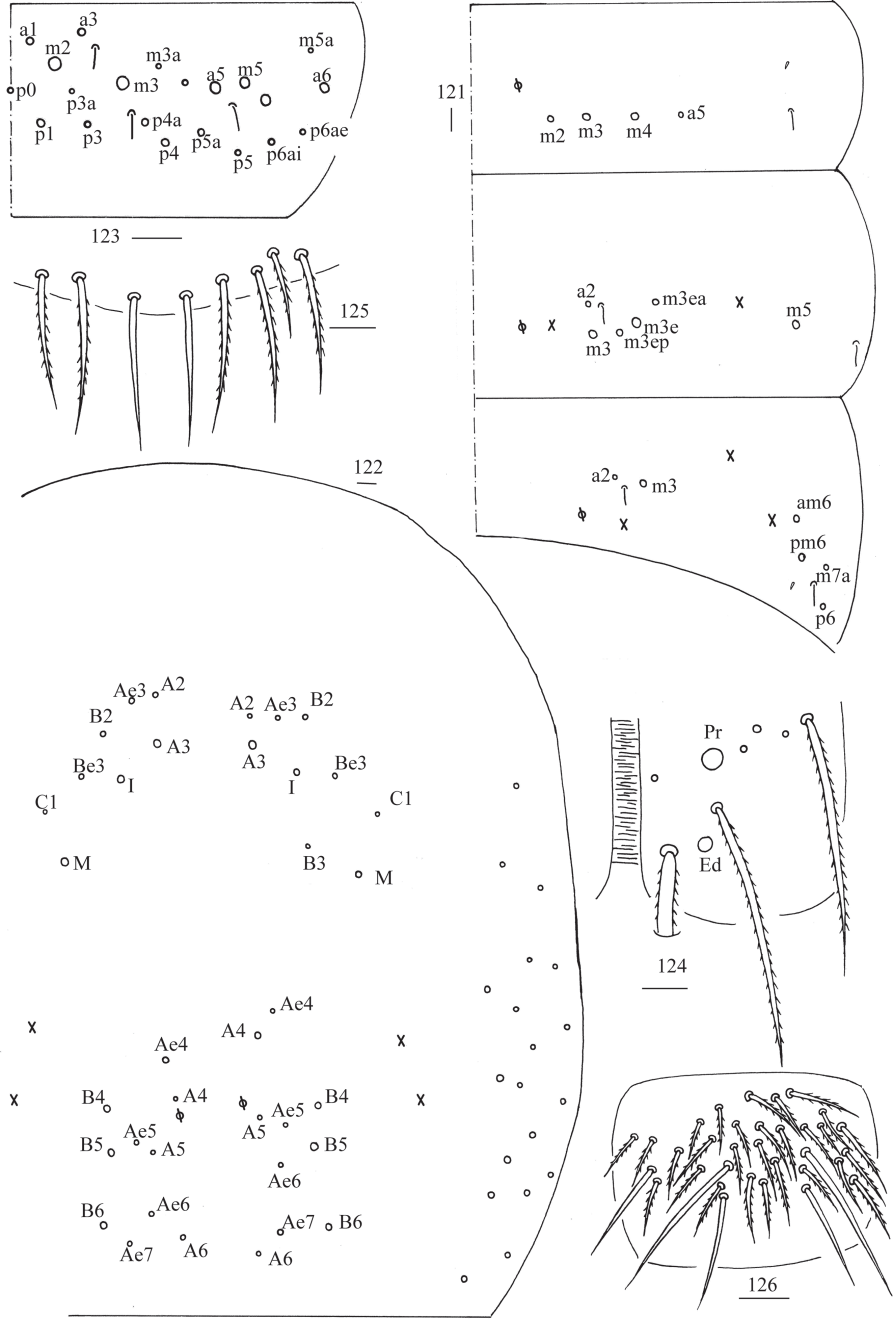
*Lepidosira montis* sp. nov. **121**. chaetotaxy of Abd. I–III (right side); **122**. chaetotaxy of Abd. IV (right side and left side partially); **123**. chaetotaxy of Abd. V (right side); **124**. anterior face of ventral tube distally; **125**. posterior face of ventral tube apically; **126**. lateral flap of ventral tube. Scale bars: 20 μm.

**Figures 127–129.**
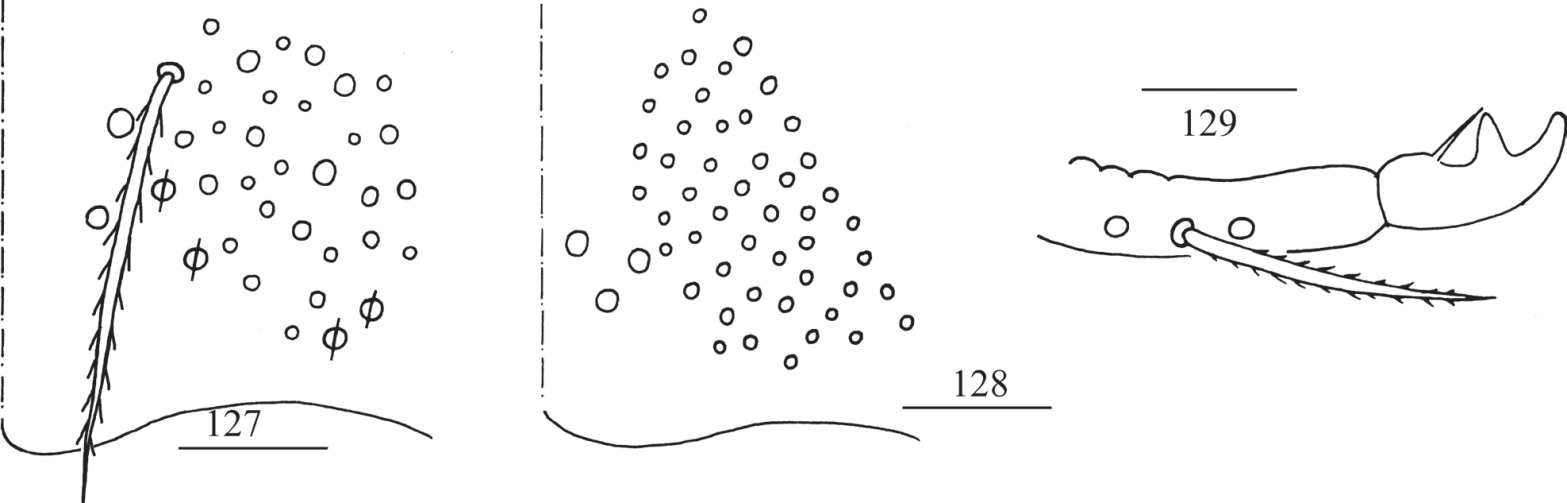
*Lepidosira montis* sp. nov. **127**. manubrial plaque (dorsal view); **128**. ventro-apical part of manubrium; **129**. mucro (lateral view). Scale bars: 20 μm.

***Abdomen***: Range of Abd. IV length as 5.50–7.86 times dorsal axial length of Abd. III. Tergal ms formula on Abd. I–Abd. V as 1, 0, 1, 0, 0, sens as 1, 2, 2, 2, 3. Abd. I with four (m_2_, m_3_, m_4_, a_5_) mac, ms anterior to sens. Abd. II with five (a_2_, m_3_, m_3e_, m_3ea_, m_3ep_) central, one (m_5_) lateral mac. Abd. III with two (a_2_, m_3_) central, four (am_6_, pm_6_, m_7a_, p_6_) lateral mac (Fig. 121). Abd. IV with two normal sens, 17–19 central and 19–21 lateral mac (Fig. 122). Abd. V with three sens (Fig. 123). Anterior face of ventral tube scaled with 3+3 large and many small ciliate chaetae, line connecting proximal and external-distal mac oblique to median furrow (Fig. 124); posterior face scaled with two apical smooth chaetae besides numerous ciliate chaetae in different size (Fig. 125); each lateral flap with five smooth and 22–28 ciliate chaetae (Fig. 126). Manubrial plate dorsally with 32–34 ciliate mac and 4–6 pseudopores (Fig. 127); ventrally with 44–51 ciliate chaetae (Fig. 128). Mucro bidentate; tip of basal spine reaching apex of subapical tooth; distal smooth section of dens almost equal to mucro in length (Fig. 129).

##### Etymology.

The type locality, Chongqing Municipality, is a mountainous region and the Latin montis for mountain.

##### Ecology.

Found in litter of subtropical forest, mainly composed of leaves of *Buxus sinica*, *Ilex yunnanensis*, *Pinus armandi*, and *Rosa corymbulosa*.

##### Remarks.

The two new species, *L. guilinensis* sp. nov. and *L. montis* sp. nov., are very similar in the colour pattern of the trunk, but almost the whole antenna is brown pigmented in *L. guilinensis* sp. nov. and only the distal Ant. III is brown pigmented in *L. montis* sp. nov. They are also somewhat similar to the Vietnamese species *L. nigropunctata* (Nguyen, 2005) and the New Zealand species *L. terraereginae* (Ellis & Bellinger, 1973) in colour pattern, but their differences are great, such as mac on Th. III and Abd. I and scales on ventral tube (Table 4).

#### Genus Willowsia

Taxon classificationAnimaliaEntomobryomorphaEntomobryidae

Shoebotham, 1917

B5D9F517-2655-512F-A38F-1C23423BD7C0

##### Type species.

*Seira nigromaculata* Lubbock, 1873: 146.

#### Willowsia zhangi sp. nov.

Taxon classificationAnimaliaEntomobryomorphaEntomobryidae

4EF1D4CB-DD74-5675-A5C1-831975F47F79

https://zoobank.org/FD29BC37-0FB0-4A9E-8981-B544D62A7415

Figs 130, 131, 132, 133, 134, 135, 136, 137, 138, 139, 140, 141, 142, 143, 144, 145, 146, 147, 148, 149, 150, 151, 152, 153, 154, 155, 156, 157, 158, 159, 160, Tables 2, 5

##### Type material.

***Holotype*** • ♀ on slide, China, Chongqing Municipality, Wuxi County, Yintiaoling National Nature Reserve, Hongqi Protection Station, 22-VII-2024, 31°30'32"N, 109°49'10"E, 1129.1 m asl, sample number 1310. ***Paratypes*** • 3♀♀ on slides, same data as holotype. All collected by Y-T Ma.

**Table 5.** Main differences between *W. zhangi* sp. nov. and similar species.

Characters	*W. zhangi* sp. nov.	*W. nigromaculata*	*W. guangdongensis*
Scale type	spinulate	long basal rib	long basal rib
Posterior mac on Th. II	16–20	7	3
Central mac on Th. III	8	2	1
Mac on Abd. I	3	3	2
Central mac on Abd. II	4	3	3
Central mac on Abd. III	3	3	2

##### Description.

***Size***: Body length up to 2.50 mm.

**Figures 130–131.**
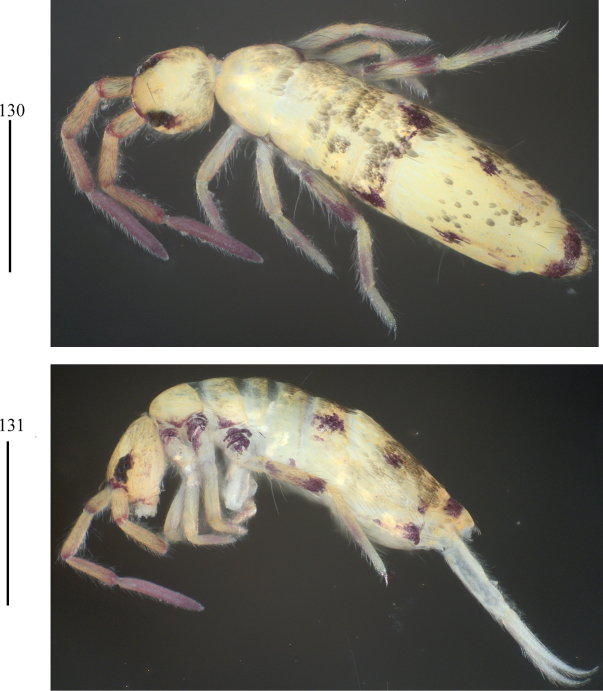
Habitus of *Willowsia zhangi* sp. nov. (**130**. dorsal view; **131**. lateral view). Scale bar: 1 mm.

***Colour pattern***: Ground colour pale yellow. Eye patches dark blue; basal part of Ant. I, distal part of each segment of antenna, coxae, and femur of hind leg with brown pigment; Abd. III and IV with an irregular brown spot sublaterally. Abd. V with a brown stripe posteriorly (Figs 130, 131).

**Figures 132–137.**
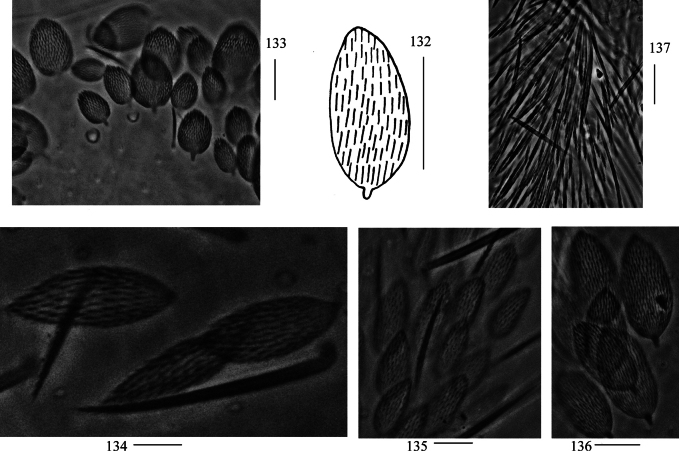
*Willowsia zhangi* sp. nov. **132**. scale (dorsal view); **133**. photomicrograph of scales on terga (dorsal view); **134**. photomicrograph of scales on Ant. I–II (dorsal view); **135**. photomicrograph of scales on leg; **136**. photomicrograph of scales on ventral manubrium; **137**. photomicrograph of chaetae on ventral dens. Scale bars: 20 μm.

***Scales***: Scales spinulated type (Fig. 132), present on terga (Fig. 133), Ant. I–II (Fig. 134), head, legs (Fig. 135), ventral side of manubrium (Fig. 136). Ant. III–IV, ventral tube, and dentes without scales (Fig. 137).

**Figures 138–145.**
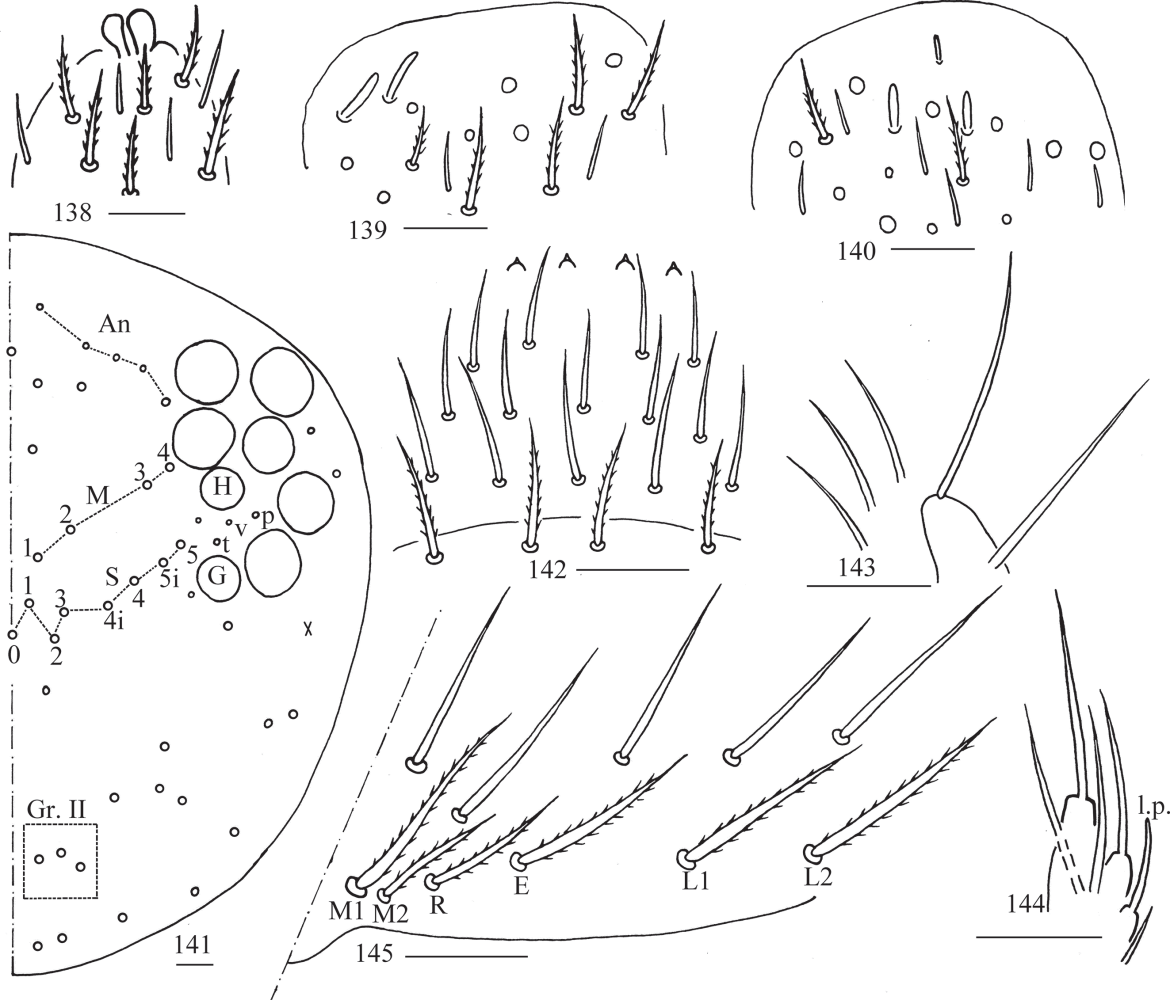
*Willowsia zhangi* sp. nov. **138**. apex of Ant. IV (dorsal view); **139**. distal Ant. III (ventral view); **140**. distal Ant. II (ventral view); **141**. dorsal head (right side); **142**. prelabrum and labrum (dorsal view); **143**. maxillary palp and outer lobe (right side); **144**. labial papilla E (right side); **145**. labial and post-labial chaetotaxy (right side). Scale bars: 20 μm.

***Head***: Antenna not annulated and 0.52–0.55 times length of body. Ratio of Ant. I–IV as 1.00/1.43–2.00/1.33–1.93/2.00–3.07. Distal part of Ant. IV with many sensory chaetae and normal ciliate chaetae, apical bulb bilobed (Fig. 138). Sensory organ of Ant. III with two rod-like chaetae (Fig. 139). Ant. II with 2–3 rod-like sensilla apically (Fig. 140). Eyes 8+8, G and H smaller than others, interocular area with p, v, t setae. Dorsal chaetotaxy of head with 5–7 antennal (An), four median (M), eight sutural (S) mac and three mac in Gr. II (Fig. 141). Prelabral and labral chaetae as 4/5, 5, 4, prelabral chaetae ciliate and other smooth, labral papillae conical (Fig. 142). Basal chaeta on maxillary outer lobe almost as thick as apical one; sublobal plate with three long smooth chaetae-like processes (Fig. 143). Lateral process of labial papilla E differentiated, with tip not reaching apex of papilla E (Fig. 144). Labial base with M_1_M_2_REL_1_L_2_, all ciliate (Fig. 145).

**Figures 146–151.**
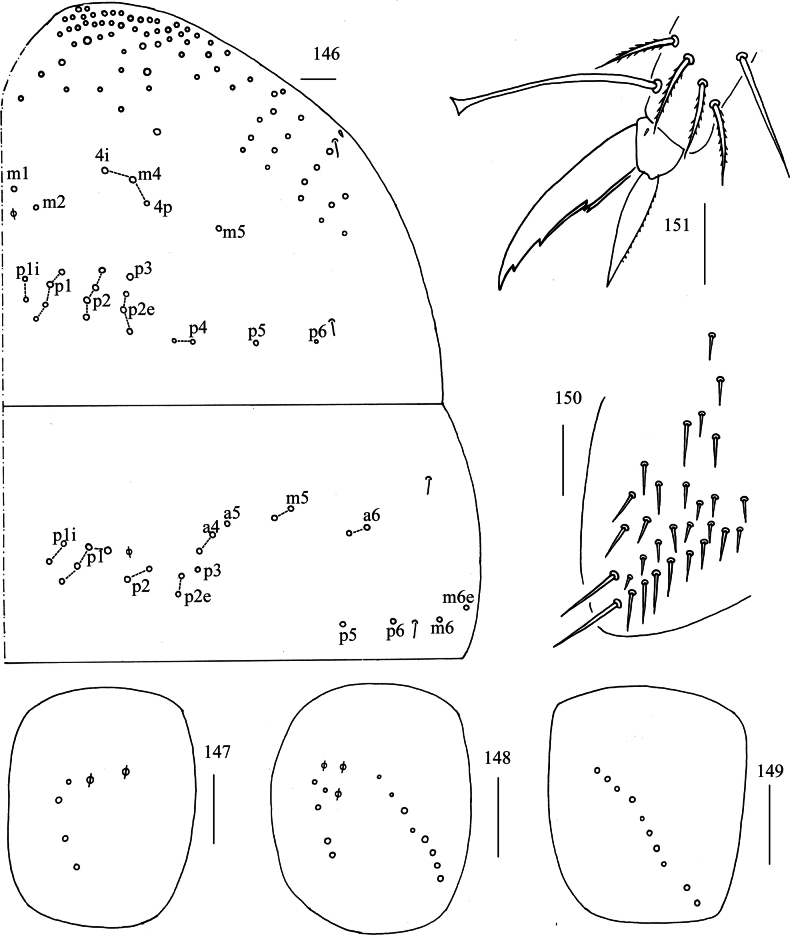
*Willowsia zhangi* sp. nov. **146**. chaetotaxy of Th. II–III (right side); **147–149**. coxal chaetotaxy of fore, middle and hind leg; **150**. trochanteral organ (ventral view); **151**. hind foot complex (lateral view). Scale bars: 20 μm.

***Thorax***: Th. II with two medio-medial (m_1_, m_2_), three medio-sublateral (m_4_, m_4i_, m_4p_), 16–20 posterior mac, one ms and two sens. Th. III with eight central and 14 lateral mac, two sens (Fig. 146). Coxal chaetal formula as 4–5/5, 8–9/9–10 (Figs 147–149). Trochanteral organ with 28–58 smooth chaetae (Fig. 150). Tenent hair clavate, 1.09–1.20 length of inner edge of unguis; unguis with four inner teeth, basal pair located at 0.32–0.38 distance from base of inner edge of unguis, distal teeth at 0.67–0.70 and 0.87–0.88 distance from base, respectively; unguiculus lanceolate, outer edge slightly serrate (Fig. 151).

**Figures 152–155.**
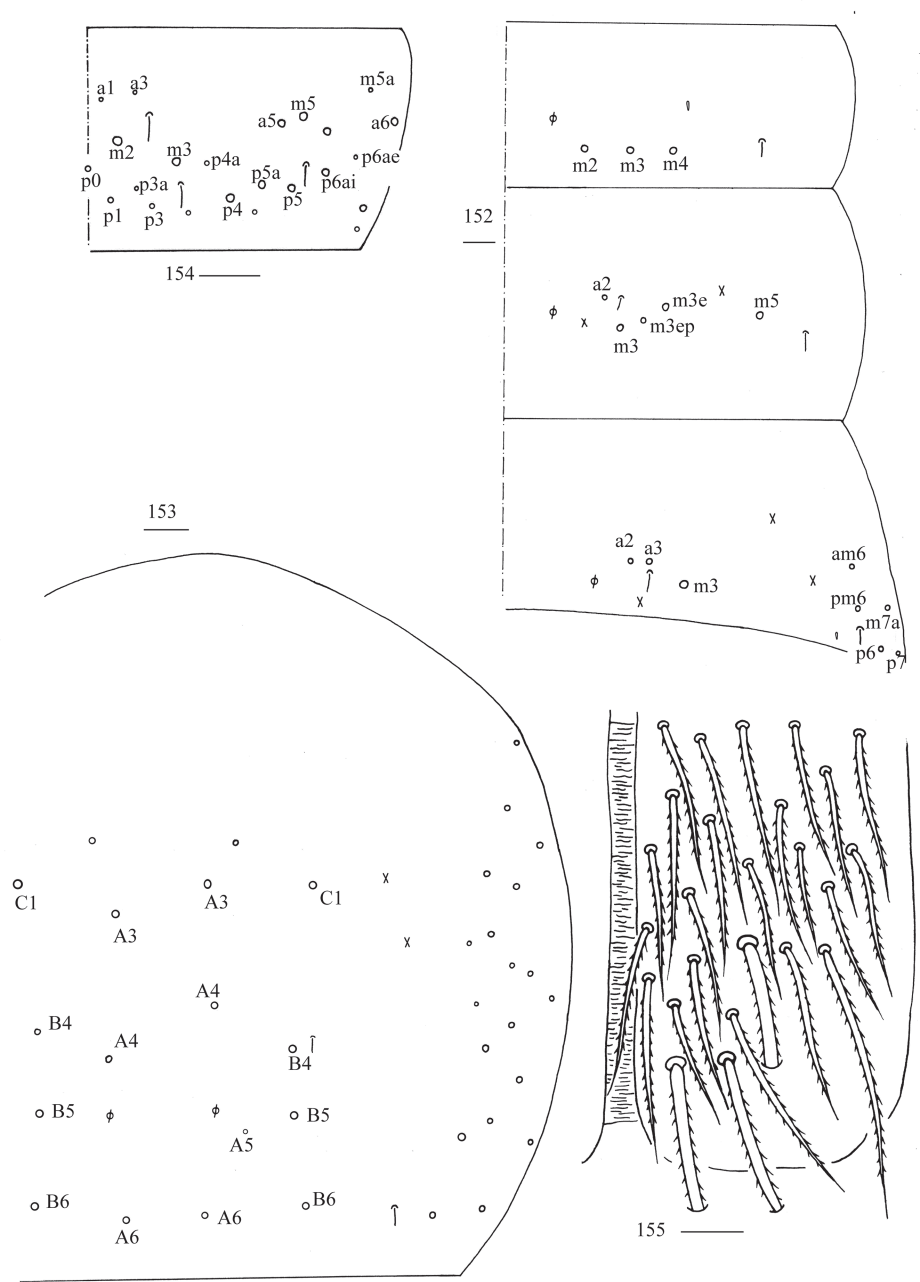
*Willowsia zhangi* sp. nov. **152**. chaetotaxy of Abd. I–III (right side); **153**. chaetotaxy of Abd. IV (right side and left side partially); **154**. chaetotaxy of Abd. V (right side); **155**. anterior face of ventral tube. Scale bars: 20 μm.

**Figures 156–160.**
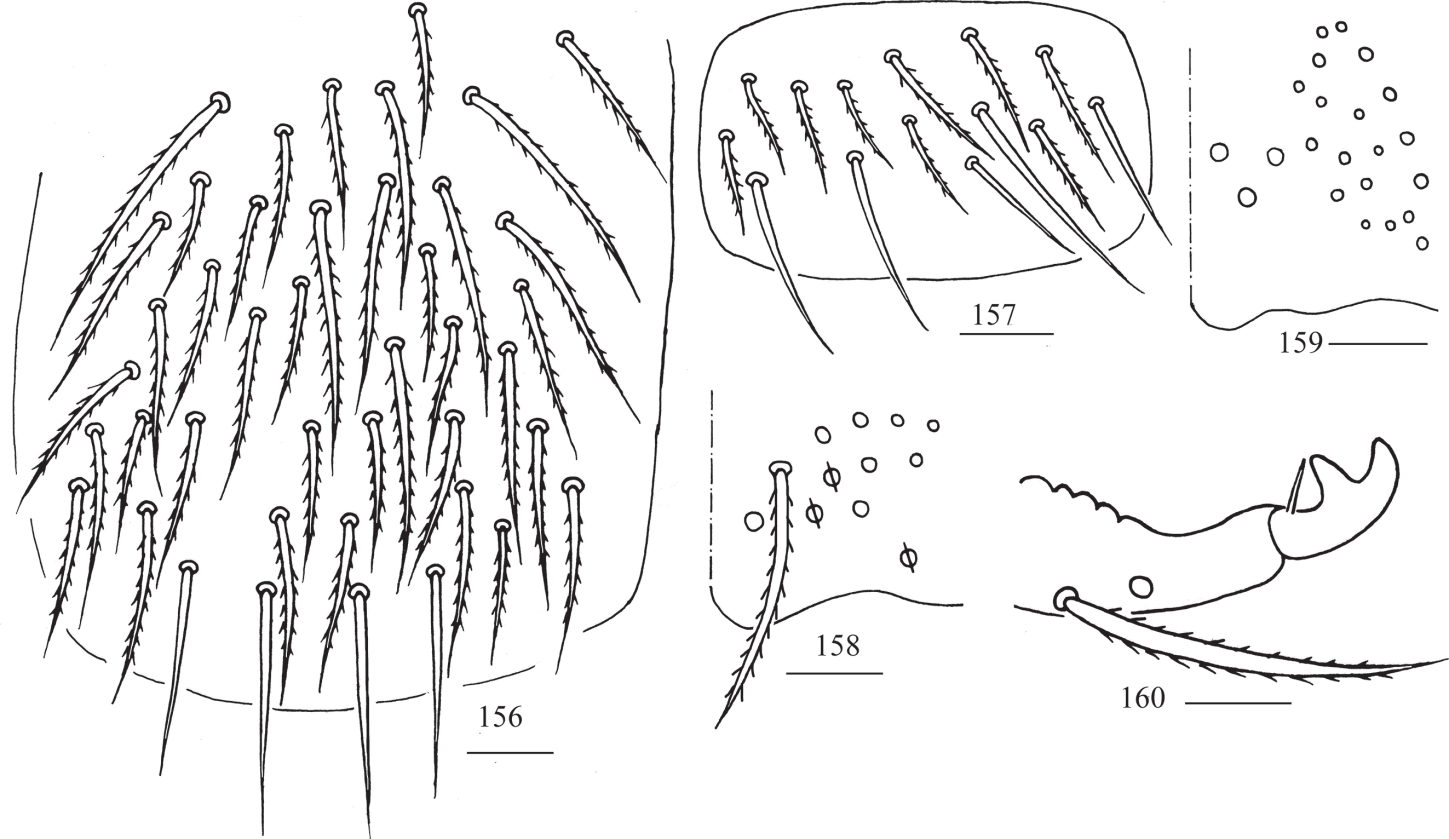
*Willowsia zhangi* sp. nov. **156**. posterior face of ventral tube; **157**. lateral flap of ventral tube; **158**. manubrial plaque (dorsal view); **159**. ventro-apical part of manubrium; **160**. mucro (lateral view). Scale bars: 20 μm.

***Abdomen***: Range of Abd. IV length as 3.57–6.36 times dorsal axial length of Abd. III. Tergal ms formula on Abd. I–Abd. V as 1, 0, 1, 0, 0, sens as 1, 2, 2, 2, 3. Abd. I with 3(4) (m_2_, m_3_, m_4_, a_5_ rarely present) mac, ms inner to sens. Abd. II with four (a_2_, m_3_, m_3e_, m_3ep_) central, one (m_5_) lateral mac. Abd. III with three (a_2_, a_3_, m_3_) central, five (am_6_, pm_6_, m_7a_, p_6_, p_7_) lateral mac (Fig. 152). Abd. IV with two normal sens, 8–9 central and 16–21 lateral mac (Fig. 153). Abd. V with three sens (Fig. 154). Anterior face of ventral tube with 3+3 large and many small ciliate chaetae, line connecting proximal and external-distal mac oblique to median furrow (Fig. 155); posterior face with two (rarely 4) apical smooth chaetae besides numerous ciliate chaetae in different size (Fig. 156); each lateral flap with 4–5 smooth and 9–12 ciliate chaetae (Fig. 157). Manubrial plate dorsally with 7–12 ciliate mac and three pseudopores (Fig. 158); ventrally with 12–22 ciliate chaetae (Fig. 159). Mucro bidentate; tip of basal spine reaching apex of subapical tooth; distal smooth section of dens almost equal to mucro in length (Fig. 160).

##### Etymology.

Named after Prof. Zhisheng Zhang from Southwest University, China, who helped to collect the specimens of this species.

##### Ecology.

Found in leaf of subtropical litter, mainly composed of leaves of *Buxus sinica*, Dendrobenthamia japonica var. chinessis, *Ilex yunnanensis*, *Rosa corymbulosa*, and *Viburnum betulifolium*.

##### Remarks.

The new species is similar to the common species *W. nigromaculata* (Lubbock, 1873) in Lubbock 1873 and the Chinese species *W. guangdongensis* Zhang, Xu & Chen, 2007 in Zhang et al. 2007 in colour pattern, but their scale type and chaetotaxy of the body differ (Table 5).

## Discussion

**Figures 161–169.**
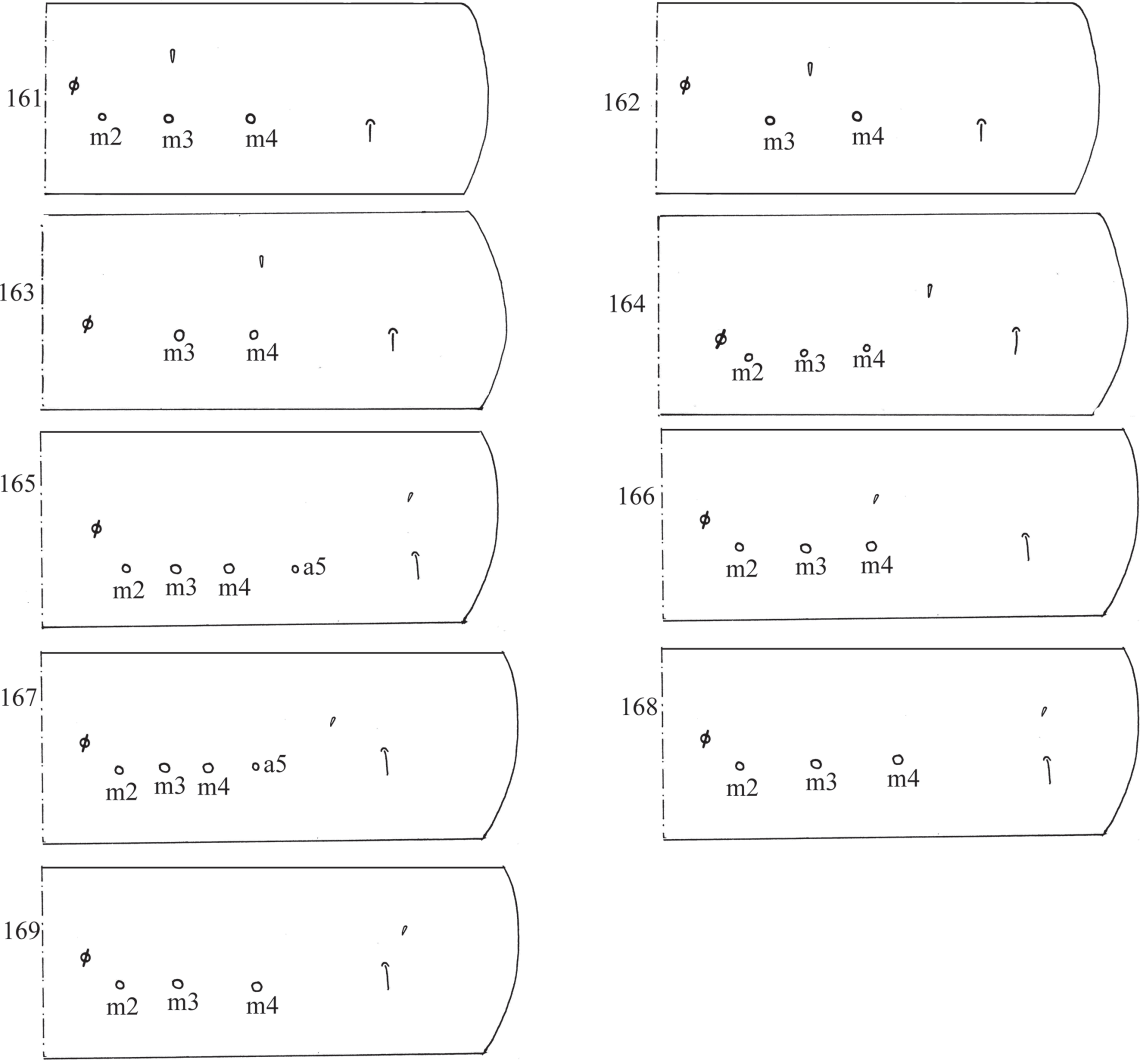
Chaetotaxy of Abd. I; **161**. *Lepidodens maculata* sp. nov.; **162**. *Lepidodens taishunensis* Lin, Wu & Pan, 2024; **163**. *Lepidosira cheni* sp. nov.; **164**. *Lepidosira guilinensis* sp. nov.; **165**. *Lepidosira montis* sp. nov.; **166**. *Willowsia pseudobartkei* Zhou, Pan & Ma, 2021.; **167**. *Willowsia christianseni* Chang & Ma, 2018; **168**. *Willowsia japonica* (Folsom, 1897); **169**. *Willowsia sexchaeta* Chang & Ma, 2018.

The colour pattern and body chaetotaxy are two main characters in the traditional taxonomy of Collembola, and the S-chaetae are increasingly used because of their intraspecific stability. There are two types of S-chaetae: one is specialized microchaetae (ms) and the other is specialised ordinary chaetae (sens). In Entomobryidae, there is one ms and one sens on Abd. I. The position of the sens is relatively stable, but the position of the ms on Abd. I varies greatly intraspecifically (Figs 161–169).

Among the six species of *Lepidodens*, the ms on Abd. I is anterior (Fig. 161) or slightly outer (Fig. 162) to the m_3_mac. Judging from the little variation of the position of the ms on Abd. I and the same type (the basal long rib type) of scales, it seems that the genus *Lepidodens* is monophyletic.

Although there are 57 known species in *Lepidosira*, the S-chaetae were mentioned only in one species: *L. neotropicalis* Nunes & Bellini, 2019 (Nunes et al. 2019). Among the three new species of *Lepidosira* and *L. neotropicalis*, the scales are of the spinulate type, but the ms on Abd. I is anterior (Fig. 163) or outer (Fig. 164) to the m_4_mac or anterior to the sens (Fig. 165). Further research is needed to determine whether the genus *Lepidosira* is monophyletic.

In *Willowsia*, the morphology of scales differs intraspecifically and the ms on Abd. I is anterior (Fig. 166) or outer (Fig. 167) to the m_4_mac or anterior (Fig. 168) or outer (Fig. 169) to the sens. It appears that the genus *Willowsia* is polyphyletic, which was confirmed by Zhang et al. (2014).

### Key to the species of the genus *Lepidodens*

**Table:** 

1	Abd. I–III with dark pigment almost entirely	**2**
–	Abd. I–III without or with only a little dark pigment	**3**
2	Th. II and Abd. IV without dark pigment	***L. nigrofasciatus* Zhang & Pan, 2016**
–	Th. II and Abd. IV with dark pigment	***L. similis* Zhang & Pan, 2016**
3	Abd. I with two mac	***L. taishunensis* Lin, Wu & Pan, 2024**
–	Abd. I with one or three mac	**4**
4	Head with three sutural mac	***L. hainanicus* Zhang & Pan, 2016**
–	Head with six sutural mac	**5**
5	Th. II with eight central mac	***L. huadingensis* Guo & Pan, 2022**
–	Th. II with four central mac	***L. maculata* sp. nov**.

## Supplementary Material

XML Treatment for Lepidodens

XML Treatment for Lepidodens maculata

XML Treatment for Lepidosira

XML Treatment for Lepidosira cheni

XML Treatment for Lepidosira guilinensis

XML Treatment for Lepidosira montis

XML Treatment for Willowsia

XML Treatment for Willowsia zhangi

## Additional information

### Use of AI

No use of AI was reported.

### Author ORCIDs

Xiao-Wei Qian https://orcid.org/0000-0002-5642-9995

Yi-Tong Ma https://orcid.org/0000-0002-8660-0503

### Data availability

All of the data that support the findings of this study are available in the main text.
